# Effectiveness of a systematic home-based albuminuria screening programme to detect chronic kidney disease in high-risk individuals in primary care (SALINE): a cross-sectional screening study

**DOI:** 10.1016/j.eclinm.2025.103185

**Published:** 2025-04-08

**Authors:** Dominique van Mil, Lyanne Marriët Kieneker, Evelien Harms, Grietje Harmanna Prins, Iris van Geer-Postmus, Maaike Mepschen, Marika Teresa Leving, Nilouq Stoker, Jan Willem Herman Kocks, Ronald Teunis Gansevoort, Hiddo Jan Lambers Heerspink

**Affiliations:** a Department of Clinical Pharmacy and Pharmacology, University Medical Center Groningen, University of Groningen, Groningen, the Netherlands; b Division of Nephrology, Department of Internal Medicine, University Medical Center Groningen, University of Groningen, Groningen, the Netherlands; c General Practitioners Research Institute, Groningen, the Netherlands; d Observational and Pragmatic Research Institute, Singapore, Singapore; e Groningen Research Institute Asthma and COPD (GRIAC), University of Groningen, University Medical Center Groningen, Groningen, the Netherlands; f Department of Pulmonology, University of Groningen, University Medical Center Groningen, Groningen, the Netherlands

**Keywords:** Chronic kidney disease, Screening, Primary care, Albuminuria

## Abstract

**Background:**

Although guidelines recommend opportunistic screening for chronic kidney disease (CKD) in individuals with established risk factors, such as diabetes, hypertension, or cardiovascular disease, screening for CKD in these individuals remains suboptimal. This study aimed to evaluate the effectiveness of a systematic home-based albuminuria screening program in primary care patients at risk for CKD.

**Methods:**

A cross-sectional screening study was performed in ten general practices and five pharmacies in the Netherlands from November 2021 to May 2024. A random selection of patients aged 45–80 years at risk for CKD based on risk factors registered in their electronic medical record was invited for home-based albuminuria screening using a urine collection device for measurement of the urinary albumin-to-creatinine ratio (ACR). In those patients with confirmed increased albuminuria (ACR ≥3 mg/mmol), an elaborate screening to assess the presence of CKD and cardiovascular risk factors was performed, followed by a referral to their general practitioner (GP) for evaluation of the findings. The primary outcome was the yield of the home-based albuminuria screening and elaborate screening to detect increased albuminuria in the GP and the pharmacy setting. SALINE is registered with ClinicalTrials.gov, NCT05321095.

**Findings:**

In total, 6380 patients (3802 via ten GPs and 2578 via five pharmacies) were invited for home-based albuminuria screening. The participation rate was 40·1% among patients invited via their GP (1524/3802), compared to 21·8% (562/2578) among those invited via their pharmacy (P < 0·001). In total, 8·7% of the GP participants had confirmed increased albuminuria (133/1524), compared to 6·0% of the pharmacy participants (34/562). Of the 115 GP participants with detected increased albuminuria who completed the elaborate screening, 102 (88·7%) were identified with one or more newly diagnosed CKD or cardiovascular risk factor(s) (n = 46, 40·0%), or with a known risk factor that was outside the target range for treatment (n = 75, 65·2%). Of the pharmacy participants with detected increased albuminuria completing the home-based screening, 26 completed the elaborate screening. Of those, 22 (84·6%) were identified with one or more newly diagnosed CKD or cardiovascular risk factor(s) (n = 6, 2·3%), or with a known risk factor that was outside the target range for treatment (n = 21, 80·8%).

**Interpretation:**

Systematic albuminuria screening of patients at risk for CKD in primary care, when performed in addition to regular opportunistic screening, has an acceptable participation rate and yield when performed via GPs, whereas it is less effective when performed via pharmacies. Such a screening program identifies patients with yet unknown albuminuria who may benefit from starting or optimizing kidney and cardioprotective treatment. The introduction of such systematic albuminuria screening programs via GPs merits further study to optimize the participation and yield.

**Funding:**

This study is funded by AstraZeneca Netherlands.

**Research in context**

**Evidence before this study**

Early detection for chronic kidney disease (CKD) is required to treat individuals with CKD early and reduce the risk of complications. Systematic screening of high-risk populations could provide an opportunity to detect those with CKD early, as currently guideline-recommended opportunistic screening practices are not sufficient. We searched PubMed with the terms “chronic kidney disease”, “albuminuria”, “targeted screening”, “opportunistic screening”, “diabetes”, “hypertension”, and “cardiovascular disease”, for articles published between Jan 1, 2000, and Jan 1, 2024, in all languages, to identify studies investigating systematic screening programs to improve guideline adherence for targeted CKD screening. Although it is apparent from systematic reviews that targeted systematic screening may be effective, there is a wide variety in how screening is performed, with the majority of studies relying on single eGFR (estimated glomerular filtration rate) measurements. There are no studies investigating the participation rate and effectiveness of a targeted systematic screening by measuring albuminuria in a home-based setting to detect early CKD.

**Added value of this study**

This cross-sectional screening study is, to our knowledge, the first to assess the effectiveness of home-based albuminuria screening among primary care patients at risk for CKD. We invited patients with risk factors for CKD for home-based albuminuria screening. Patients with confirmed increased albuminuria were invited for an elaborate screening for CKD and cardiovascular risk factors, followed by a referral to their general practitioner for evaluation of the findings. The study shows that systematic home-based albuminuria among patients invited through general practitioners (GPs) has a higher participation rate (40·1%) than among patients invited through pharmacies (21·8%). Among GP practices, 8·7% of participants had confirmed increased albuminuria, with 40·6% of these cases being newly diagnosed. In pharmacy patients, the rate was 6·0%. Among those GP patients who underwent detailed screening, most patients had risk factors, either newly identified or previously known, but outside the recommended treatment ranges. Of these individuals, 52·9% sought further evaluation from their GP, resulting in treatment changes for 44·4% of them.

**Implications of all the available evidence**

The present study shows the value of systematic, targeted home-based albuminuria screening in primary care, favouring a general practitioner setting over a pharmacy setting. The results underline the importance of guideline optimalization and need for systematic albuminuria screening on top of opportunistic, regular evaluation of albuminuria in high-risk patients. Future studies should investigate the improvement of participation and implementation of care following screening.

## Introduction

Chronic kidney disease (CKD) is recognized as a major public health problem with an increasing prevalence that currently exceeds 10% of the global adult population.^1^^,^^2^ CKD is associated with a high risk of morbidity and mortality due to kidney failure requiring kidney replacement therapy and cardiovascular complications.^3^ The increasing CKD prevalence is mainly attributable to the increase in prevalence of risk factors for CKD, such as obesity, diabetes, hypertension, and cardiovascular disease (CVD). Therefore, early disease recognition and monitoring of individuals at risk for CKD is required to avoid an unnecessary delay in the start of preventive treatment and reduce the risk of complications associated with CKD.

The diagnosis of CKD relies on the assessment of the glomerular filtration rate (GFR), as measure of the kidney function, and albuminuria, as measure of the degree of kidney damage.^4^ Currently, international diabetes, cardiovascular and CKD-guidelines recommend regular assessment of both the estimated GFR (eGFR) and albuminuria in patients at risk as targeted, opportunistic CKD screening.^4^, ^5^, ^6^, ^7^ However, worldwide, the implementation of CKD screening remains suboptimal in these high-risk populations.^8^, ^9^, ^10^ The assessment of albuminuria is especially low, varying between 4 and 40%, depending on the patient population and country. This implies that opportunistic CKD screening alone may not be sufficient.

The therapeutic options for kidney protection in CKD have expanded in recent years. This is of particular relevance since these therapies, such as sodium-glucose cotransporter 2 (SGLT2) inhibitors, the non-steroid mineralocorticoid receptor antagonist (ns-MRA) finerenone, and the glucagon-like peptide 1 (GLP1) agonist semaglutide, have shown not only to slow CKD progression in early stages of CKD, but also to prevent CVD. These benefits on both kidney and cardiovascular outcomes appear to be at least partially mediated by the early reduction in albuminuria.^11^, ^12^, ^13^, ^14^ This, along with their generally favorable safety profile, makes these drug classes valuable treatment options for patients with CKD. In addition, advances have been made with respect to albuminuria testing, facilitating the opportunity to screen for albuminuria at home.

Given these considerations, we aimed to investigate whether a systematic, targeted home-based screening for albuminuria is effective in primary care to detect individuals with yet unknown CKD.

## Methods

### Study design and population

The Screening for Albuminuria at the first LINE for early identification of chronic kidney disease (SALINE) study was a cross-sectional screening study performed in ten general practices and five pharmacies, in different parts of the Netherlands to ensure generalisability across the country, between November 2021 and November 2023. Follow-up data was collected until May 2024. The study consisted of a home-based screening for albuminuria and subsequent elaborate screening of individuals with confirmed increased albuminuria to assess the presence of CKD and cardiovascular risk factors, followed by a referral to their general practitioner (GP) for evaluation of the findings.

Patients were selected for invitation for the home-based screening based on their age (45–80 years) and having risk factors for CKD progression. These patients were registered at the participating general practices or pharmacies and randomly selected through an electronic health platform used by the general practices and pharmacies, that can perform searches through the electronic medical records. Patients in the GP-group were identified from the GPs’ electronic medical records based on the following risk factors: type 1 and type 2 diabetes (T1D or T2D, respectively), cardiovascular disease, hypertension, lipid disorder, or obesity. Risk factors were identified in the electronic medical records using International Classification of Primary Care (ICPC) codes (Supplementary Table S1). Patients in the pharmacy-group were identified based on drug prescriptions for the aforementioned risk factors in the last six months. Drug prescriptions were identified in the pharmacy databases using Anatomical Therapeutic Chemical (ATC) codes (Supplementary Table S1). After this identification process, a random subset of eligible patients was invited for home-based screening. Participants were enrolled in the study if they returned the signed informed consent form.

### Procedures

For the home-based albuminuria screening, the selected patients received a standardized invitation letter from their GP or pharmacist. This included a participant information folder and informed consent form, a questionnaire and urine test. The questionnaire addressed sociodemographic details (age, sex) and medical details (height, weight, smoking status, self-reported medical history concerning cardiovascular risk factors and albuminuria). Socioeconomic status (SES) was assessed using the socioeconomic status district score from the participating GP or pharmacist, provided by Statistics Netherlands. The urine test consisted of a urine collection device (UCD, the CE-marked PeeSpot test) and instructions. Participants were instructed to collect midstream urine from an early morning void. Subsequently, the participant was asked to send the signed informed consent form, completed questionnaire, and UCD by mail. In this urine sample, albumin and creatinine concentrations were measured by the laboratory of the University Medical Center Groningen (UMCG) by immunoturbidimetry and an enzymatic method, respectively. The urinary albumin-to-creatinine ratio (ACR) was assessed based on the Kidney Disease Improving Global Outcomes (KDIGO) categorization (normal [A1; <3 mg/mmol], or moderately/severely increased [A2/A3; ≥3 mg/mmol]). If the first measurement showed increased albuminuria (e.g., albuminuria category A2 or A3), participants were sent a second urine test for confirmation. If the result of the second test was indicative of normal albuminuria (e.g., albuminuria category A1), a third test was sent. If the first increased albuminuria result was confirmed by either the second or third test, the participant was considered to have increased albuminuria and invited for an elaborate screening for CVD and CKD risk factors. For the participants of the GP-group, a confirmational test was not required if a recent (meaning, within one year prior to the home-based screening) measurement performed by the participants' GP also indicated increased albuminuria. This previous measurement would then count as the confirmational test of a participant's first increased home-based urine test in the current study.

During the elaborate screening performed by study personnel at the participants' general practice or pharmacy, CKD and cardiovascular disease risk factors were measured using a questionnaire, physical examination, and point of care (POC) measurements. The questionnaire addressed smoking status, medical history of kidney disease, albuminuria, T1D or T2D, cardiovascular disease, hypertension and lipid disorders, and medication usage over the last six weeks. Physical examination entailed measurement of height, weight, blood pressure, and pulse. POC measurements in capillary blood were performed to determine the HbA1c and creatinine using the Abbott Afinion 2™ and Abbott i-STAT Alinity, respectively. The eGFR was estimated based on the creatinine levels using the Chronic Kidney Disease Epidemiology Collaboration (CKD-EPI) 2009 equation.^4^ After the elaborate screening, a report was sent to the participant and the participant's GP by post, based on the screening findings. To determine the presence of risk factors, the definitions of the prevailing national and international CKD and CVD guidelines were used (Supplementary Table S2).^4^^,^^15^, ^16^, ^17^ The report sent to the GP included a recommendation for treatment and follow-up care based on the presence of risk factors in the individual involved and the prevailing guidelines. If no risk factors were found besides increased albuminuria, the participant and GP were recommended to perform yearly CKD and CVD screening as recommended by the prevailing guidelines.

Follow-up data collection of participants was performed within six months after the elaborate screening visit and only possible for the GP participants. The individual GPs of the pharmacy patients were not themselves participating in the study and follow-up data from their patients could therefore not be retrieved. The GPs were approached to confirm whether a consultation with the referred participant had taken place and whether the screening findings and treatment recommendations had led to the initiation of diagnostic tests and adjustments to treatment (including lifestyle and cardiometabolic drug prescriptions).

### Outcomes

The primary outcome was the participation rate and yield of the home-based albuminuria screening and the elaborate screening, assessed separately for the screening groups (GP-group and pharmacy-group) and compared between the screening groups. The participation rate was defined as the number of individuals completing the home-based screening relative to the number of individuals invited. The yield of the home-based screening was defined as the number of individuals who had confirmed increased albuminuria during the home-based screening relative to the number of individuals participating. The yield of the elaborate screening was defined as the number of individuals with increased albuminuria that was identified with newly diagnosed risk factors for CKD or cardiovascular disease or risk factors that were known but not meeting treatment targets.

Secondary outcomes were the characteristics of the participants; the yield of the home-based albuminuria screening in terms of previously unknown albuminuria and persistent albuminuria; and the GP follow-up rate within six months and rate of changes in lifestyle treatment and cardiometabolic drug prescriptions. The yield in terms of previously unknown albuminuria was defined as the number of individuals with increased albuminuria, that was not previously recorded in the electronic medical records of the GP (GP-group only) in the previous year. This includes individuals with previously unmeasured increased albuminuria and individuals with previous ACR measurements <3 mg/mmol. The yield of persistent albuminuria was defined as the number of individuals with increased albuminuria despite receiving albuminuria-lowering treatment with RAS-inhibitors. Additionally, among the GP patients eligible for home-based albuminuria screening, the compliance to yearly albuminuria screening according to the prevailing guidelines was assessed and defined as an albuminuria measurement recorded in the 18 months.

### Statistical analysis

Descriptive statistics were used to describe the data. Categorical variables are reported using numbers with proportions. Continuous variables are summarized using mean (SD) or median (IQR). Differences between groups for categorical data were tested with a χ^2^ test; differences between groups for continuous data were tested by Student's t-test or a Mann–Whitney test in case of skewed distribution. Normality was assessed by a histogram of the data, a Q–Q plot and using the Shapiro–Wilk test. To assess the participation rate across subgroups of SES of the GPs and pharmacies, the socioeconomic status district score was assessed in tertiles to categorize low, middle, and high SES using a χ^2^ test for comparisons, as well as a multivariate linear regression adjusting the participation rate for the study group (GP/pharmacy) and SES tertile. The statistical significance level was set at P-values of less than 0·05. IBM SPSS (version 28) was used for all analyses. SALINE was a feasibility study with multiple study outcomes. Therefore, no formal sample size calculation was performed. It was chosen not to impute any missing data concerning medical history of the participants, due to the limited amount of missing data. No data was missing for the participants of the elaborate screening. SALINE is registered with ClinicalTrials.gov, NCT05321095.

### Ethics

The study protocol was approved by the medical research ethics committee *Beoordeling Ethiek Biomedisch Onderzoek (BEBO)* Foundation (Assen, the Netherlands; 21·139/ck). All participants provided written informed consent before participation in the home-based screening and elaborate screening.

### Role of the funding source

This study is funded by AstraZeneca Netherlands. The funders of the study provided support and advice in the preparation and execution of the study, but were not involved in decisions regarding the study design, data collection, data analysis, interpretation, writing of the report, or the decision to submit.

## Results

From November 2021 to August 2023, patients from ten general practices and five pharmacies were screened for eligibility. Of all eligible patients from the general practices, 38·8% did not have an ACR measurement recorded in the electronic medical record in the previous 18 months. Between the ten practices this non-measurement rate ranged from 16·6% to 60·5% (Supplementary Table S3). Of the remaining 61·2% of whom an ACR measurement from the previous 18 months was available, 12·4% had increased albuminuria (ACR ≥3 mg/mmol), and 87·6% had no increased albuminuria (ACR <3 mg/mmol). Based on the selected ICPC codes, 27·3%, 13·2%, 35·3%, 55·4%, and 30·6% of the eligible patients from the general practices had a recorded diagnosis of diabetes, obesity, lipid disorders, hypertension, and CVD, respectively (Supplementary Table S3). Characteristics of the eligible pharmacy patients could not be explored due to the methods for screening for eligibility in the electronic medical records: the search would select patients based on drug prescriptions, but if a patient was described a certain drug, the selection system would not further search for other relevant drug prescriptions. A random subset of 3802 eligible patients from the general practices and 2578 eligible patients from the pharmacies was invited for home-based albuminuria screening. In general, invited GP patients had a higher district SES compared to invited pharmacy patients (Supplementary Table S5).

### Participation and results of the targeted home-based screening

**Fig. 1:**
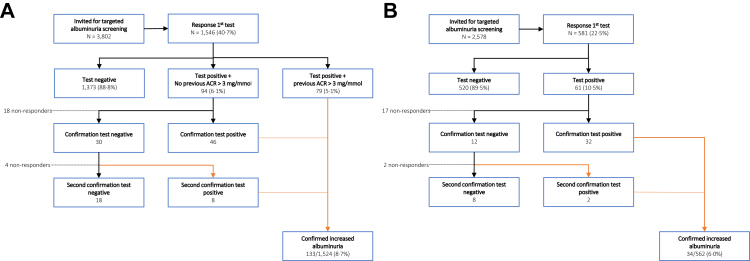
**Participation rate and yield of home-based albuminuria screening in ten general practices (A) and five pharmacies (B)**.

The participation rate was 40·1% among the invited GP patients (1524 out of 3802 invited patients) and 21·8% among the invited pharmacy patients (562 out of 2578 invited patients) (P < 0·001) (Fig. 1). Adjusting the participation rates of the GP and pharmacy practices for differences in SES using a linear regression showed that both study group (pharmacy or general practice) and SES (low, middle, high) are independently associated with the participation rate. For both the GP patients and pharmacy patients, the participation rate was significantly higher in patients with a higher SES (Supplementary Table S5). Compared to the pharmacy participants, GP participants were significantly younger and were less likely to have a medical history of T2D, hypertension, a lipid disorder and obesity (Table 1).

**Table 1:** Characteristics of the GP and pharmacy patients completing the home-based screening and the patients with confirmed increased albuminuria.

Characteristics	General practices (Invited: 3·802)		Pharmacies (Invited: 2·578)		P for completing^c^	P for increased albuminuria^c^
	Patients completing home-based screening	Patients with increased albuminuria	Patients completing home-based screening	Patients with increased albuminuria		
Total, n (%)^a^	1·524 (40·2%)	133 (8·7%)	562 (21·8%)	34 (6·1%)	P < 0·001	P = 0·046
Age, years; mean (SD)	64·8 (10·5)	69·8 (9·2)	66·6 (9·2)	69·1 (10·2)	P < 0·001	P = 0·68
Men, n (%)	786 (51·6%)	93 (69·9%)	294 (52·7%)	23 (67·6%)	P = 0·56	P = 0·80
Current smokers, n (%)	186 (12·2%)	28 (21·1%)	69 (12·5%)	5 (14·7%)	P = 0·74	P = 0·65
*Socioeconomic status district score*. n (%)						
Low	133 (8·7%)	17 (12·8%)	285 (50·7%)	19 (55·9%)	P < 0·001	P < 0·001
Middle	516 (33·9%)	54 (40·6%)	149 (26·5%)	7 (20·6%)		
High	875 (57·4%)	62 (46·6%)	128 (22·8%)	8 (23·5%)		
*Self-reported medical history, n (%)*^d^						
Diabetes type 1	33 (2·2%)	1 (0·8%)	12 (2·2%)	0	P = 0·96	P = 0·61
Diabetes type 2	173 (11·5%)	46 (34·6%)	92 (16·5%)	13 (38·2%)	P = 0·0021	P = 0·69
Hypertension	749 (49·6%)	95 (71·4%)	351 (62·9%)	28 (82·4%)	P < 0·001	P = 0·20
Cardiovascular disease	342 (22·6%)	57 (42·9%)	127 (22·8%)	15 (44·1%)	P = 0·96	P = 0·89
Lipid disorder	621 (41·1%)	97 (72·9%)	264 (47·3%)	24 (70·6%)	P = 0·012	P = 0·78
Obesity	177 (11·7%)	14 (10·5%)	46 (8·2%)	5 (14·7%)	P = 0·024	P = 0·49
*Albuminuria status as measured by the general practitioner within the previous year, n (%)*						
ACR >3 mg/mmol	108 (7·1%)	79 (59·4%)	–	–	–	–
ACR <3 mg/mmol	441 (28·9%)	8 (6·0%)	–	–	–	–
ACR not measured	824 (54·1%)	39 (29·3%)	–	–	–	–
ACR status unavailable^b^	151 (9·9%)	7 (5·3%)	–	–	–	–

Data are mean (SD) or n (%).

a (%) for patients completing home-screening from total invited, % for patients with increased albuminuria from completing the home-based screening.

b For one general practice, no distinction could be made between ACR <3 mg/mmol and ACR not measured, resulting in these patients being classified as ACR status unavailable.

c Screening via general practices vs screening via pharmacies.

d (%) Responses to the questionnaire for medical history are lacking for 14 GP patients and 4 pharmacy patients completing the home-based screening. ACR = albumin-to-creatinine ratio.

Among the GP participants, 8·7% had confirmed increased albuminuria (133 out of 1524 patients) (Fig. 1, Table 1). Of those patients, 40·6% (54 out of 133 patients) had previously unknown albuminuria, according to their electronic medical records. Increased albuminuria was confirmed in 6·0% of the pharmacy participants (34 out of 562 patients).

### Elaborate screening

In total, 115 of the 133 GP patients (86·5%) invited for the elaborate screening participated in this screening. The majority of these participants were male, and their mean age was 69·1 years (SD 9·4) (Table 2). In 58·3% we identified persistent albuminuria despite RAAS-inhibiting therapy (66 out of 115 patients), and only 5·2% (6 out of 115) and 3·5% (4 out of 115) were using an SGLT-2 inhibiting drug or GLP-1 agonist, respectively. The median ACR was 7·1 mg/mmol (IQR 4·6–12·9), and in 94% (108 out of 115 patients), albuminuria was moderately increased (ACR 3–30 mg/mmol). More than 30% of those with known increased albuminuria according to their medical record, reported being unaware of having increased albuminuria prior to the screening.

**Table 2:** Characteristics and identified risk factors of the GP participants with increased albuminuria as measured during elaborate screening.

	Participants (n = 115)^c^
**Characteristics**	
Age, years (mean, SD)	69·1 (9·4)
Men (n (%))	80 (69·6%)
Current smokers (n (%))	26 (22·6%)
BMI, kg/m2 (mean, SD)	28·6 (4·8)
Obesity (n (%))	40 (34·8%)
History of cardiovascular disease (n (%))	52 (45·2%)
History of hypertension (n (%))	85 (73·9%)
Use of antihypertensive drugs (n (%))	88 (76·5%)
Use of RAAS inhibiting drugs (n (%))	67 (58·3%)
Systolic blood pressure, mm Hg (mean, SD)	142·3 (19·4)
Diastolic blood pressure, mm Hg (mean, SD)	82·8 (10·7)
History of type 1 diabetes (n (%))	0 (−)
History of type 2 diabetes (n (%))	40 (34·8%)
Use of glucose-lowering drugs (n (%))	36 (31·3%)
Use of SGLT-2 inhibiting drugs (n (%))	6 (5·2%)
Use of GLP-1 agonist (n (%))	4 (3·5%)
HbA1c, mmol/mol (mean, SD)	45·3 (13·6)
History of impaired kidney function (n (%))	13 (11·3%)
eGFR, ml/min/1·73 m^2^ (mean, SD)	74·1 (21·0)
eGFR <60 ml/min/1·73 m^2^ (n (%))	31 (28·2%)
History of increased albuminuria (ACR ≥3 mg/mmol recorded in previous year) (n (%))	69 (60·0%)
Self-reported awareness of having increased albuminuria (n (%))	47 (68·1%)
ACR, during home-based screening (median, IQR)	7·1 (4·6–12·9)
*ACR category (*n (%))	
ACR 3–30 mg/mmol	108 (93·9%)
ACR >30 mg/mmol	5 (4·3%)
*CKD KDIGO stage by prognosis*^a^*(*n (%))	
Moderately increased risk	77 (70·6%)
High risk	18 (16·5%)
Very high risk	14 (12·8%)
**Identified risk factors for CKD and CVD (**n (%))	
Individuals with one or more newly diagnosed risk factor or known, outside target treatment range^b^	102 (88·7%)
Individuals with one or more newly diagnosed risk factor^b^	46 (40·0%)
Individuals with one or more risk factor known, outside target treatment range^b^	75 (65·2%)
Presence of decreased kidney function (eGFR <60 ml/min/1.73 m^2^), total	31 (28·2%)
Decreased kidney function, newly diagnosed	23 (20·9%)
Decreased kidney function, known	8 (7·3%)
Hypertension, total	108 (93·9%)
Hypertension, newly diagnosed	24 (20·9%)
Hypertension, known within target treatment range	18 (15·7%)
Hypertension, known outside target treatment range	66 (57·4%)
Diabetes, based on HbA1c, total	43 (37·4%)
Diabetes, newly diagnosed	3 (2·6%)
Diabetes, known within target treatment range	23 (20·0%)
Diabetes, known outside target treatment range	17 (14·8%)
Prediabetes during screening and no history of diabetes, based on HbA1c	29 (25·2%)

Data are n (%), mean (SD), or median (IQR).

GP = general practitioner. BMI = body mass index. RAAS = renin angiotensin aldosterone system. SGLT-2 = sodium glucose co-transporter-2. HbA1c = hemoglobin A1c; eGFR = estimated glomerular filtration rate. ACR = albumin-to-creatinine ratio. CKD = chronic kidney disease. CVD = cardiovascular disease.

a Based on one single eGFR-measurement.

b Taking into account the risk factors decreased kidney function, hypertension, diabetes.

c Serum creatinine values and eGFR measurements were missing for five participants; ACR values are missing for two participants: the CKD KDIGO stages and presence of decreased kidney function are n (%) from the available number of patients with available data.

**Fig. 2:**
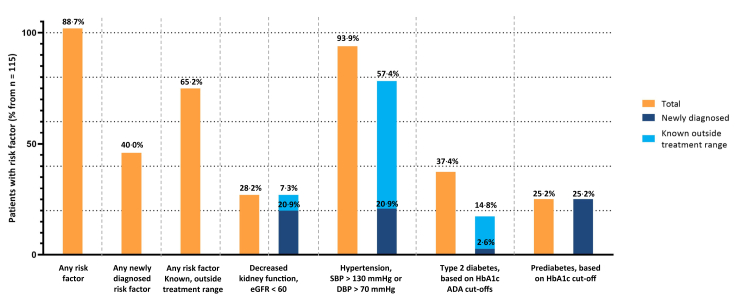
**Identified risk factors in GP patients in the elaborate screening**. eGFR: estimated glomerular filtration rate; SBP: systolic blood pressure; DBP: diastolic blood pressure; HbA1c: hemoglobin A1c.

In 102 participants (88·7%) of 115 participants, one or more risk CKD and/or CVD factors were found that were either newly diagnosed (n = 46, 40·0%) or known, but outside target treatment range (n = 75, 65·2%) (Table 2, Fig. 2). Hypertension was the most commonly diagnosed risk factor: among 24 (20·9%) and 66 (57·4%) of 115 participants, respectively, hypertension was newly diagnosed or known but not meeting treatment targets. In 17 patients known with T2D, the HbA1c levels were not meeting treatment targets. Additionally, based on the HbA1c levels, T2D and prediabetes were newly identified in 3 and 29 participants, respectively, who were not previously diagnosed with T2D (2·6% and 25·2%, respectively). 23 participants (20·9%) were newly diagnosed with a decreased kidney function (eGFR <60 ml/min/1·73 m^2^).

The results of the elaborate screening of the pharmacy patients are shown in the supplementary material (Supplementary Table S6). In summary, 26 of the 34 pharmacy patients (76·5%) invited for the elaborate screening participated in this screening, of whom the mean age was 68·0 (SD 10·6). Persistent albuminuria despite RAAS-inhibiting therapy was found in 76·9% (20 out of 26 patients). In 84·6% (22 out of 26 patients), albuminuria was moderately increased. In total, 22 patients (84·6%) were identified with one or more newly diagnosed CKD or cardiovascular risk factor(s) (n = 6, 2·3%), or with a known risk factor that was outside the target range for treatment (n = 21, 80·8%). Among 23 patients with known hypertension, the blood pressure was not meeting treatment targets in 20 of them. T2D and prediabetes were, according to the HbA1c levels, newly diagnosed in 2 (7·7%) and 4 (15·4%) patients, respectively.

### Implementation of GP care

Follow-up data was available for all GP participants referred to their GP following the elaborate screening visit (102 out of 102) (Table 3). Of those participants, 54 (52·9%) had a consultation with their GP to evaluate the screening findings. The GP repeated diagnostic tests in 45 participants (83·3%) and changed treatment in 24 (44·4%) of those participants consulting their GP. This included lifestyle treatment in 14 participants (25·9%) and changes in cardiometabolic drug prescriptions in 13 participants (24·1%).

**Table 3:** Implementation of care of GP participants of the elaborate screening after referral to general practitioner.

	Participants (n = 102)
**Consultation with GP after elaborate screening visit**	54 (52·9%)
**Performance of repeat diagnostic tests**	45 (83·3%)
Blood pressure	42 (77·8%)
Glucose/HbA1c	32 (59·3%)
Lipid profile	28 (51·9%)
eGFR	31 (57·4%)
ACR	30 (55·6%)
**Initiation or change in treatment**	24 (44·4%)
Lifestyle interventions	14 (25·9%)
Cardiometabolic pharmacotherapy	13 (24·1%)
Blood pressure lowering drugs^a^	7 (13·0%)
Cholesterol lowering drugs	3 (5·6%)
Glucose lowering drugs^b^	4 (7·4%)
**Referral to nephrologist**	3 (5·6%)

Data are n (%).

GP = general practitioner. HbA1c = hemoglobin A1c. eGFR = estimated glomerular filtration rate. ACR = albumin-to-creatinine ratio.

a An RAAS-inhibitor was prescribed in two patients, a diuretic was prescribed in one patient, a calcium-channel blocker was prescribed in three patients, and the type of prescribed blood pressure lowering drug was unknown in one patient.

b An SGLT-2 inhibitor was prescribed in three patients and a GLP1-agonist was prescribed in one patient.

## Discussion

In this cross-sectional screening study assessing the effectiveness of home-based albuminuria screening among patients from primary care at risk for CKD, we observed a real-world albuminuria testing rate of 61·2%. The participation rate among the 6380 individuals invited for our home-based albuminuria screening program was higher among patients invited by GPs compared to patients invited by pharmacies, with 40·1% vs 21·8% of the invited patients, respectively. Among the GP practices, confirmed increased albuminuria was found in 8·7% of the participants, with 40·6% of these individuals being newly diagnosed. Confirmed increased albuminuria was found in 6·0% of the participating pharmacy patients. Of the 115 GP participants who completed the elaborate screening, only 58·3% used RAAS-inhibiting therapy, and 5·2% used SGLT2 inhibitors. Most of the patients also had risk factors that were either newly diagnosed or previously known, but outside target treatment ranges. Of these individuals, 52·9% consulted their GP for further evaluation as recommended. In 44·4% of them, this consultation led to a change in treatment.

The finding that nearly 40% of the eligible patients, who are at risk of CKD, had no recorded ACR measurement within the past 18 months aligns with previous research. Two studies on CKD screening adherence among US patients with type 2 diabetes reported ACR testing rates of 43·3% and 52·3%.^9^^,^^10^ A lower screening rate of 5·1% was found in non-diabetic patients with hypertension. Similarly, a global meta-analysis found ACR testing rates over two years of 35·1% in patients with diabetes and 4·1% in patients with hypertension.^8^ This underscores the variability of the already limited albuminuria screening rates for different risk factors of CKD, which may be influenced by discrepancies between guidelines. Specifically, the KDIGO 2024 guideline prioritizes CKD screening in patients with diabetes, CVD, and hypertension.^4^ The American Diabetes Association (ADA) recommends screening for CKD by the eGFR and ACR beginning at the time of diagnosis of T2D and five years after diagnosis of T1D, and to repeat testing annually.^6^ Guidelines for the management of hypertension and cardiovascular risk offer less clarity on CKD screening. For instance, the European Society of Cardiology (ESC) 2021 CVD prevention guideline considers opportunistic CKD screening for eGFR and albuminuria in all individuals in whom cardiovascular risk estimation is indicated. The ESC 2024 hypertension guideline recommends ACR screening for all individuals with hypertension without mentioning a repeat interval, to be repeated annually in patients with CKD.^5^^,^^7^ In contrast, the International Society of Hypertension 2020 guideline recommends screening by dipstick urine analysis.^18^ This shows that these guidelines vary regarding their recommendations in whom to screen for CKD and often lack specific recommendations regarding the timing and frequency of screening. Nevertheless, our findings confirm the notion that opportunistic CKD screening alone may not be sufficient for early detection of CKD in high-risk patients.

Our systematic screening program targeting high-risk individuals, added to opportunistic screening, showed that GP patients had a 40·1% participation rate in the home-based albuminuria screening compared to 21·8% of pharmacy patients. This participation rate is limited compared to a previous screening study that we performed in the general population, which found a participation rate of 59% for home-based albuminuria screening.^19^ This suggests that individuals are more inclined to respond to an invitation for general population screening than to an invitation from a healthcare provider. Several barriers and facilitators that have previously been identified for health checks for cardiometabolic diseases in primary care, may explain the uptake of the current study compared to higher uptake of population screening. Characteristics such as smoking, lower SES, and having more cardiovascular risk factors have been related to non-participation in cardiometabolic health checks, whereas higher SES and being among the “worried well” are linked to higher participation.^20^ Unfamiliarity with CKD and unawareness of CKD risk may also contribute to low participation rates.^21^ Further research is needed to better understand the reasons for (non)participation compared to population screening and to increase public awareness of CKD. A recent review by Tuot and Plantinga summarizes strategies how awareness of CKD can be increased.^22^ Attention by policy makers was thought to be essential to stimulate the development of awareness campaigns. Despite community pharmacists being accessible healthcare providers for high-risk CKD patients, the lower participation among pharmacy patients suggests that invitations from GPs are more effective at motivating patients to engage in CKD screening. This is in keeping with the findings related to the uptake of cardiometabolic health checks, that being invited by the GP and the often longstanding relationships of patients with GPs may make patients responsive to their invitation.^20^

Among the participants of this study, increased albuminuria was identified in 8·7% of the GP patients and 6·0% of the pharmacy patients. These prevalences are higher than those reported in previous general population studies in the Netherlands but lower than global rates in high-risk populations.^10^^,^^19^^,^^23^^,^^24^ This difference may reflect improvements in screening and treatment in the Netherlands compared to other parts of the world. Notwithstanding, our study indicates that a substantial proportion (40·6%) of individuals with increased albuminuria remain undiagnosed. Notably, nearly 90% of the GP participants with increased albuminuria also had one or more CVD or CKD risk factors that were newly diagnosed or known but did not meet treatment targets. Hypertension management was often suboptimal, and many patients were not receiving RAAS-inhibiting therapy despite the presence of increased albuminuria. Therefore, this study identified patients in whom health benefit can be obtained by installing guideline-recommended therapies. The use of newer albuminuria-lowering therapies, such as SGLT-2 inhibitors, was even lower. Moreover, while almost 90% of the GP patients with increased albuminuria attended the elaborate screening visit, only 53% followed our recommendation to consult their GP to evaluate the screening findings and to consider start or change in treatment, which was done in only 44% of the participants who did consult their GP. These relatively low rates of follow-up and treatment change need additional study. However, the findings may suggest that guideline adherence in CKD management is limited, consistent with previous studies highlighting deficiencies in CKD care, including suboptimal monitoring, failure to reach blood pressure targets, and insufficient rates of prescription of guideline-recommended therapies.^25^, ^26^, ^27^, ^28^ Additionally, many patients are unaware of the severity of having a CKD diagnosis and its consequences for morbidity and mortality.^21^^,^^29^ This may prevent them from consulting their GP despite recommendations to do so. Patient awareness could be improved by targeting health systems, leading to improved quality of CKD care in primary care settings end improved CKD diagnosis and subsequently increased awareness among patients.^22^ However, time constraints, high workload, and the perception of CKD as an abstract and complex concept, currently inhibit GPs from performing opportunistic screening and monitoring, and delivering optimal CKD care, combined with the experience that CKD guidelines are insufficiently aligned with clinical practice in primary care and poorly integrated into cardiovascular risk programs.^30^^,^^31^ Improving follow-up and implementation of CKD management is necessary for CKD screening programs to have optimal effect. An alternative approach that could improve identification, screening and subsequent treatment of patients with CKD is the use of an automatic clinical decision support system (CDS). Such a system generates automatic alerts in the electronic medical record when delivered medical care is not optimal according to guidelines. These CDS appear to have promising effects when investigated as an intervention targeting specifically CKD, in a research setting.^32^^,^^33^ In the Netherlands such a decision support system exists not only for CKD, but for all major medical domains. Our results should therefore be interpreted as addition to a CDS. Systematic reviews show that the efficacy of clinical decision support systems in general and specifically aimed at CKD is only moderate, among others by alert fatigue.^34^^,^^35^ Due to the same alerts for many patients or many alerts for one patient, physicians tend to ignore or even turn-off the alert systems. Another possibility to improve the implementation of care could be the development of screen-and-treat facilities, where screening for risk factors is performed, followed by the prescription of treatment according to prevailing guidelines. Such facilities could optimize risk management for the patient without increasing the burden on GPs and is an area for further research.

Several strengths of this study can be highlighted. To our knowledge, this is the first targeted, systematic CKD screening using home-based albuminuria testing. By including both pharmacies and GP practices, the study reflects a broad primary care setting. The diverse range of participating practices across the Netherlands, including various socioeconomic backgrounds, ensured a representative study population. It is well known that the burden of CKD varies between counties due to geographic, economic and social factors.^36^^,^^37^ Screening studies worldwide are therefore required to evaluate the screening intervention across other healthcare systems. Our study also has limitations. The lack of data from non-participants in the home-based screening phase, due to privacy legislation that prohibited us from collecting data from non-participants, limits our understanding of the characteristics of non-participants and factors influencing decisions to participate. Importantly, having had a previous ACR measurement could influence the decision to participate and thus the participation rate. The ACR status of both non-participants and of pharmacy patients was unknown, as this is not recorded in the pharmacy medical records, not enabling us to perform a sensitivity analysis adjusting for those individuals with known previous testing. Data regarding medical history of the participants of the home-based screening relied on self-reporting, which creates a possible bias. Moreover, the first step of the screening for CKD did not include eGFR screening but only albuminuria screening. However, this has limited consequences because, as shown by previous studies, eGFR measurement is already better implemented in CKD screening of high-risk patients than albuminuria.^9^ Additionally, individuals with decreased eGFR without increased albuminuria are at lower risk of progression of CKD and CVD compared to those with increased albuminuria, and treatment benefits appear higher in those with higher albuminuria levels compared to those with lower levels or without increased albuminuria. This supports the role of albuminuria as first target of screening.^4^^,^^22^^,^^38^^,^^39^ Finally, the study design did not allow for assessment of indicators of test accuracy (sensitivity, specificity, predictive values). However, test accuracy was analyzed in a previous study showing good test characteristics for the applied UCD-method (sensitivity 96·6%, specificity 97·3%) partly caused by the confirmation tests that are incorporated that reduce the likelihood of false-positive results.^19^

In summary, the results of the present study provide valuable insights into the current practice regarding opportunistic albuminuria screening in high-risk patients and the effectiveness of an additional systematic, home-based albuminuria screening in primary care. Real-world albuminuria testing in high-risk primary care patients appeared to be suboptimal, with a substantial proportion of patients not being screened for albuminuria as recommended by guidelines. Our study underlines the importance of guideline optimization and the need for more awareness for screening and regular evaluation of albuminuria in high-risk patients. Although targeted systematic albuminuria screening in a pharmacy appears to offer little added benefit due to the low participation rate and the low number of patients identified with increased albuminuria, screening in a GP setting shows benefits on top of opportunistic screening. In those patients identified with increased albuminuria, a large part had previously undiagnosed or sub-optimally controlled risk factors, suggesting that health benefits can be obtained by initiating or optimizing treatment of CKD risk factors. Nevertheless, the participation rate and implementation of care following screening need to be improved.

## Contributors

DvM, GHP, IvG-P, JWHK, RTG, and HJLH conceptualized the study underlying the present manuscript. DvM, LMK, EH, GHP, IvG-P, MM, MTL, NS, JWHK, RTG, and HJLH were involved in the conduct of the study underlying the present manuscript. DvM, LM, MM, and HJLH performed the analysis of the data. DvM, RTG, and HJLH wrote the original draft of the manuscript. LMK, EH, GHP, IvG-P, MM, MTL, NS, and JWHK reviewed and edited the manuscript. DvM, Eh, GHP, IvG-P, NS, and HJLH directly accessed and verified the underlying data, and all authors took the responsibility for the decision to submit for publication.

All authors read and approved the final version of the manuscript.

## Data sharing statement

De-identified and anonymized participant data used in the study are available upon reasonable request to the corresponding author, approval by the joint investigators, and with a signed data access agreement.

## Declaration of interests

Professor Kocks reports grants, personal fees and non-financial support from AstraZeneca, grants, personal fees and non-financial support from Boehringer Ingelheim, grants and personal fees from Chiesi Pharmaceuticals, grants, personal fees and non-financial support from GSK, grants and personal fees from Novartis, grants from MundiPharma, grants from TEVA, outside the submitted work. He is the owner of GPRI and has stock options for 3% in Lothar MedTec. In the past three years, HJLH has received fees for consultancy or grants, or both, for research from AstraZeneca, Bayer, Boehringer Ingelheim, Chinook, CLS Pharma, Dimerix, Eli-Lilly, Fresenius, Gilead, Janssen, Novo Nordisk, and Travere Therapeutics. In the past three years, RTG has received fees for consultancy or grants, or both, for research from AbbVie, AstraZeneca, Baxter, Bayer, Healthy.io, Roche, and Sandoz. The other authors don't report any conflict of interest.

## Appendix A Supplementary data

Supplementary Tables

Research Protocol
