# Supplementary material for: Effectiveness of a systematic home-based albuminuria screening programme to detect chronic kidney disease in high-risk individuals in primary care (SALINE): a cross-sectional screening study
Source: eClinicalMedicine. 2025 Apr 8;82:103185. doi: 10.1016/j.eclinm.2025.103185 (PMC12005226; doi:10.1016/j.eclinm.2025.103185)
Supplement: Supplementary Tables [file mmc1.docx]

**Supplement to: Effectiveness of a systematic home-based albuminuria screening program in high-risk individuals in primary care to detect chronic kidney disease: the SALINE study**

Dominique van Mil, Lyanne M. Kieneker, Evelien Harms, Grietje H. Prins, Iris van Geer-Postmus, Maaike Mepschen, Marika T. Leving, Nilouq Stoker, Janwillem W.H. Kocks , Ron T. Gansevoort, Hiddo J.L. Heerspink

**Table of Contents**

**2** Table S1. Applied ICPC and ATC codes for identification of patients eligible for SALINE based on CKD and CVD risk factors

**3** Table S2. Definitions used for the determination of the presence of risk factors during the elaborate screening visit, according to prevailing national guidelines for Dutch General practitioners and international guidelines.

**4** Table S3. Differences between the patient populations of the ten general practices regarding the selected risk factors for CKD progression, and recorded ACR measurements

**5** Table S4A. Characteristics of the GP patients completing the home-based screening, per general practice

Table S4B. Characteristics of the pharmacy patients completing the home-based screening, per pharmacy

**6** Table S5. Comparison of socioeconomic status district score of invited and participating patients.

**7** Table S6. Characteristics of the pharmacy participants with increased albuminuria as measured during elaborate screening.

**9** References

**Table S1. Applied ICPC and ATC codes for identification of patients eligible for SALINE based on CKD and CVD risk factors**

| Indication | GP-group: ICPC codes | Pharmacy group: ATC codes |
| --- | --- | --- |
| Diabetes | T90·01  T90·02 | A10B (glucose-lowering drugs, excluding insulin)  A10X (other drugs used in diabetes, including aldose reductase inhibitors)  A10A (insulin and analogues) |
| Hypertension | K86  K87 | C09A, C09B (ace-inhibitors)  C09C, C09C (angiotensin receptor blockers)  C03 (diuretics)  C08 (calcium antagonists)  C07, excluding C07AA05 (beta blockers, excluding propranolol) |
| Lipid disorder | T93 | C10AA, C10BA, C10BX (statins)  C10AZ09 (ezetimibe) |
| Obesity | T82 | NA |
| Established cardiovascular disease |  |  |
| Acute coronary syndrome | M80 | NA |
| Angina pectoris | K74 | NA |
| Coronary revascularization/coronary sclerosis | Surgery | NA |
| Transient cerebral ischemic attack or stroke | K89  K90 | NA |
| Aorta aneurysm | K99·01 | NA |
| Intermittent claudication | K92·01 | NA |
| Peripheral revascularization | Surgery | NA |
| (Chronic) ischemic heart disease | K76 | NA |
| Myocardial infarction | K75 | NA |
| Atherosclerosis | K91 | NA |
| Heart failure | K77 | NA |
| ATC, anatomical therapeutic chemical; ICPC, international classification of primary care. | | |

**Table S2. Definitions used for the determination of the presence of risk factors during the elaborate screening visit, according to prevailing national guidelines for Dutch General practitioners and international guidelines.**

| Risk factor | Definition | Source |
| --- | --- | --- |
| Obesity | BMI ≥30 | NHG^1^ |
| Hypertension | SBP ≥130 mm Hg, DBP ≥80 mm Hg | NHG^1^ |
| Type 2 diabetes (newly diagnosed) | HbA1c ≥48 mmol/mol | NHG^2^, ADA^3^ |
| Type 2 diabetes (known, inadequate regulation) | HbA1c >53 mmol/mol in case of age <70 years or  HbA1c >64 mmol/mol in case of age >70 years | NHG^2^ |
| Prediabetes | HbA1c of 39-48 mmol/L | ADA^3^ |
| Impaired kidney function | eGFR <60 ml/min/1·73 m^2^ | KDIGO^4^ |
| ACR=albumin-to-creatinine ratio. ADA=American Diabetes Association. BMI=body mass index. DPD=diastolic blood pressure. eGFR= estimated glomerular filtration rate. KDIGO=kidney disease: improving global outcomes. NHG= Nederlands Huisartsen Genootschap. SBP=systolic blood pressure. | | |

**Table S3. Differences between the patient populations of the ten general practices regarding the selected risk factors for CKD progression, and recorded ACR measurements.**

|  | **Overall** | **GP1** | **GP2** | **GP3** | **GP4** | **GP5** | **GP6** | **GP7** | **GP8** | **GP9** | **GP10** |
| --- | --- | --- | --- | --- | --- | --- | --- | --- | --- | --- | --- |
| **Risk factors based on the selected ICPC codes** | | |  |  |  |  |  |  |  |  |  |
| Diabetes | 27·3% | 25·4% | 35·4% | 20·9% | 48·9% | 15·4% | 16·8% | 18·9% | 28·0% | 21·7% | 30·8% |
| Obesity | 13·2% | 23·7% | 8·6% | 3·7% | 44·9% | 6·8% | 9·9% | 8·9% | 11·4% | 6·7% | 9·0% |
| Lipid disorders | 35·3% | 30·8% | 24·9% | 41·9% | 54·2% | 51·6% | 43·8% | 37·8% | 26·4% | 17·5% | 19·8% |
| Hypertension | 55·4% | 56·9% | 50·1% | 46·1% | 47·6% | 57·8% | 66·0% | 55·6% | 63·7% | 65·2% | 51·3% |
| Cardiovascular disease | 30·6% | 24·5% | 35·9% | 27·8% | 22·6% | 27·6% | 26·8% | 40·1% | 36·1% | 31·3% | 32·3% |
| **ACR measurements recorded** |  |  |  |  |  |  |  |  |  |  |  |
| ACR not measured in previous 18 months | 38·8% | 40·2% | 41·1% | 60·5% | 23·2% | 32·7% | 49·7% | 44·8% | 35·7% | 16·6% | 38·8% |
| ACR ≥ 3 mg/mmol | 7·6% | 6·0% | 7·3% | 2·8% | 15·3% | 6·4% | 4·8% | 10·0% | 6·5% | 9·2% | 7·7% |
| ACR < 3 mg/mmol | 53·6% | 53·8% | 51·6% | 36·7% | 61·5% | 60·9% | 45·5% | 45·2% | 57·8% | 74·2% | 53·5% |

GP = general practitioner. CKD = chronic kidney disease. ACR = albumin-to-creatinine ratio. ICPC = international classification of primary care.

**Table S4A. Characteristics of the GP patients completing the home-based screening, per general practice**

|  | **GP1**  **(Invited: 464)** | **GP2**  **(Invited: 816)** | | **GP3**  **(Invited: 422)** | | **GP4**  **(Invited: 296)** | | **GP5**  **(Invited: 442)** | | **GP6**  **(Invited: 378)** | | **GP7**  **(Invited: 244)** | | **GP8**  **(Invited: 244)** | | **GP9**  **(Invited: 222)** | | **GP10 (Invited: 274)** |
| --- | --- | --- | --- | --- | --- | --- | --- | --- | --- | --- | --- | --- | --- | --- | --- | --- | --- | --- |
| Participation rate | 164 (35·3%) | 236 (28·9%) | | 191 (45·3%) | | 45 (15·2%) | | 232 (52·5%) | | 179 (47·4%) | | 88 (36·1%) | | 117 (48·0%) | | 134 (60·4%) | | 139 (50·7%) |
| **Characteristics** | | |  | |  | |  | |  | |  | |  | |  | |  | |
| Age, years; mean (SD) | 63·7 (11·6) | 60·5 (14·5) | | 64·5 (11·7) | | 60·3 (7·6) | | 65·5 (7·7) | | 66·4 (8·4) | | 65·2 (7·5) | | 65·6 (9·3) | | 69·1 (7·9) | | 66·5 (8·6) |
| Men, n (%) | 89 (54·3%) | 127 (53·8%) | | 97 (50·9%) | | 17 (37·8%) | | 126 (54·3%) | | 83 (46·4%) | | 45 (51·1%) | | 61 (52·1%) | | 69 (51·5%) | | 72 (51·8%) |
| Current smokers, n (%) | 25 (15·3%) | 42 (17·8%) | | 13 (6·8%) | | 5 (11·1%) | | 20 (8·6%) | | 18 (10·1%) | | 21 (23·9%) | | 16 (13·8%) | | 10 (7·5%) | | 16 (11·5%) |
| *Self-reported medical history, n (%)* ‡ | | | |  | |  | |  | |  | |  | |  | |  | |  |
| Diabetes type 1 | 3 (1·8%) | 6 (2·5%) | | 6 (3·1%) | | 4 (8·9%) | | 1 (0·4%) | | 1 (0·6%) | | 1 (1·1%) | | 3 (2·6%) | | 1 (0·7%) | | 7 (5·0%) |
| Diabetes type 2 | 32 (19·5%) | 27 (11·4%) | | 5 (2·6%) | | 13 (28·9%) | | 21 (9·1%) | | 14 (7·8%) | | 7 (7·9%) | | 19 (16·3%) | | 17 (12·7%) | | 18 (12·9%) |
| Hypertension | 78 (47·6%) | 99 (41·9%) | | 73 (38·2%) | | 17 (37·8%) | | 123 (53·0%) | | 97 (54·2%) | | 42 (47·7%) | | 61 (52·1%) | | 87 (64·9%) | | 72 (51·8%) |
| Cardiovascular disease | 30 (18·3%) | 72 (30·5%) | | 37 (19·4%) | | 7 (15·6%) | | 50 (21·6%) | | 26 (14·5%) | | 20 (22·7%) | | 27 (23·1%) | | 33 (24·6%) | | 40 (28·7%) |
| Lipid disorder | 51 (31·1%) | 69 (29·2%) | | 74 (38·8%) | | 23 (51·1%) | | 123 (53·0%) | | 74 (41·3%) | | 39 (44·3%) | | 50 (42·7%) | | 55 (41·0%) | | 63 (45·3%) |
| Obesity | 26 (15·9%) | 36 (15·3%) | | 8 (4·2%) | | 11 (24·4%) | | 20 (8·6%) | | 14 (7·8%) | | 9 (10·3%) | | 21 (17·9%) | | 12 (9·0%) | | 20 (14·4%) |

GP = general practitioner.

**Table S4B. Characteristics of the pharmacy patients completing the home-based screening, per pharmacy**

|  | **Pharmacy 1**  **(Invited: 566)** | **Pharmacy 2**  **(Invited: 212)** | | **Pharmacy 3**  **(Invited: 702)** | | **Pharmacy 4**  **(Invited: 450)** | | **Pharmacy 5**  **(Invited: 648)** |
| --- | --- | --- | --- | --- | --- | --- | --- | --- |
| Participation rate | 124 (21·9%) | 51 (24·1%) | | 149 (21·2%) | | 128 (28·4%) | | 110 (17·0%) |
| **Characteristics** | | |  | |  | |  | |
| Age, years; mean (SD) | 64·3 (10·2) | 67·0 (9·4) | | 67·0 (8·5) | | 68·2 (8·4) | | 64·3 (10·2) |
| Men, n (%) | 61 (49·2%) | 26 (51·0%) | | 84 (56·4%) | | 54 (42·2%) | | 69 (62·7%) |
| Current smokers, n (%) | 21 (16·9%) | 7 (13·7%) | | 10 (6·7%) | | 13 (10·2%) | | 18 (16·4%) |
| *Self-reported medical history, n (%)* ‡ | | | |  | |  | |  |
| Diabetes type 1 | 4 (3·2%) | 0 (0·0%) | | 3 (2·0%) | | 1 (0·8%) | | 4 (3·6%) |
| Diabetes type 2 | 20 (16·1%) | 8 (15·7%) | | 26 (17·5%) | | 18 (14·1%) | | 20 (18·2%) |
| Hypertension | 79 (63·7%) | 37 (72·5%) | | 101 (67·7%) | | 61 (47·7%) | | 73 (66·4%) |
| Cardiovascular disease | 24 (19·4%) | 10 (19·6%) | | 39 (26·2%) | | 27 (21·1%) | | 27 (24·5%) |
| Lipid disorder | 52 (41·9%) | 23 (45·1%) | | 64 (42·9%) | | 69 (53·9%) | | 56 (50·9%) |
| Obesity | 11 (8·9%) | 3 (5·9%) | | 11 (7·4%) | | 10 (7·8%) | | 11 (10·0%) |

**Table S5. Comparison of socioeconomic status district score of invited and participating patients.**

|  | **General practices**  **(Invited: 3·802)** | | **Pharmacies**  **(Invited: 2·578)** | |  |  |  |
| --- | --- | --- | --- | --- | --- | --- | --- |
|  | **Patients invited for home-screening (3·802)** | **Patients completing home-screening (1·524)** | **Patients invited for home-screening (2·578)** | **Patients completing home-screening (562)** | *P for invitation* * | *P for completing* * | *..* |
| **Socioeconomic status district score, n (%)** | | | | | | | |
| Lowest tertile | 540 (14·2%) | 133 (8·7%) | 1·480 (57·4%) | 285 (50·7%) | P < 0·001 | P < 0.001 |  |
| Middle tertile | 1524 (40·1%) | 516 (33·9%) | 648 (25·1%) | 149 (26·5%) |  |  |  |
| High tertile | 1738 (45·7%) | 875 (57·4%) | 450 (17·5%) | 128 (22·8%) |  |  |  |
| **Participation rate according to socioeconomic status district score** | | | | | *P for GPs vs pharmacies* | *P for tertiles of GPs* | *P for tertiles of pharmacies* |
| Lowest tertile | 133/540 (24·6%)  516/1·524 (33·9%)  875/1·738 (50·3%) | | 285/1·480 (19·3%)  149/648 (23·0%)  128/450 (28·4%) | | P < 0·05 | P < 0·001 | P < 0·001 |
| Middle tertile |  |  |  |  | P < 0·001 |  |  |
| High tertile |  |  |  |  | P < 0.001 |  |  |

Socioeconomic status (SES) was assessed using the socioeconomic status district score from the participating GP or pharmacist, meaning that every patient from one practice has been assigned to the same SES tertile. In this table, we report the results of a Chi-square test * Screening via general practices versus screening via pharmacies

**Table S6. Characteristics of the pharmacy participants with increased albuminuria as measured during elaborate screening.**

|  | **Patients**  **(n = 26)** | |
| --- | --- | --- |
| **Characteristics** |  | |
| Age, years (mean, SD) | 68·0 (10·6) | |
| Men (n (%)) | 20 (76·9%) | |
| Current smokers (n (%)) | 5 (19·2%) | |
| BMI, kg/m2 | 31·4 (6·4) | |
| Obesity (n (%)) | 14 (53·8%) | |
| History of cardiovascular disease (n (%)) | 13 (50·0%) | |
| History of hypertension (n (%)) | 23 (88·5%) | |
| Use of antihypertensive drugs (n (%)) | 24 (92·3%) | |
| Use of RAAS inhibiting drugs (n (%)) | 20 (76·9%) | |
| Systolic blood pressure, mm Hg | 143·4 (17·5) | |
| Diastolic blood pressure, mm Hg | 82·2 (13·3) | |
| History of type 1 diabetes (n (%)) | 0 | |
| History of type 2 diabetes (n (%)) | 9 (34·6%) | |
| Use of glucose-lowering drugs (n (%)) | 8 (30·8%) | |
| Use of SGLT-2 inhibiting drugs (n (%)) | 1 (3·8%) | |
| Use of GLP-1 agonist (n (%)) | 1 (3·8%) | |
| HbA1c, mmol/mol | 42·8 (10·9) | |
| History of impaired kidney function (n (%)) | 5 (19·2%) | |
| eGFR, ml/min/1.73m^2^ | 69·9 (26·0) | |
| eGFR <60ml/min/1.73m^2^ (n (%)) | 8 (30·8%) | |
| Self-reported awareness of having increased albuminuria (n, (%)) | 10 (38·5%) | |
| ACR, during home-based screening | 10·0 (4·1-17·1) | |
| ACR 3-30 mg/mmol | 22 (84·6%) | |
| ACR >30 mg/mmol | 4 (15·4%) | |
| *CKD KDIGO stage by prognosis** (n (%)) |  | |
| Moderately increased risk | 16 (61·5%) | |
| High risk | 4 (15·4%) | |
| Very high risk | 6 (23·1%) | |
| **Identified risk factors for CKD and CVD** (n (%)) |  | |
| Individuals with one or more newly diagnosed risk factor or known, outside target treatment range † | 22 (84·6%) | |
| Individuals with one or more newly diagnosed risk factor | 6 (2·3%) | |
| Individuals with one or more risk factor known, outside target treatment range† | 21 (80·8%) | |
| Presence of decreased kidney function (eGFR < 60), total | 8 (30·8%) | |
| Newly diagnosed | 4 (15·4%) | |
| Known | 4 (15·4%) | |
| Hypertension, total | 24 (92·3%) | |
| Hypertension, newly diagnosed | 1 (3·8%) | |
| Hypertension, known, within target treatment range | 3 (11·5%) | |
| Hypertension, known, outside target treatment range | 20 (76·9%) | |
| Diabetes, based on HbA1c, total | 11 (42·3%) | |
| Diabetes, newly diagnosed | 2 (7·7%) | |
| Diabetes, known, within target treatment range | 7 (26·9%) | |
| Diabetes, known, outside target treatment range | 2 (7·7%) | |
| Prediabetes during screening and no history of diabetes, based on HbA1c | 4 (15·4%) | |
| Data are n (%), mean (SD), or median (IQR). *Based on one single eGFR-measurement. † Taking into account the risk factors decreased kidney function, hypertension, diabetes. BMI = body mass index. RAAS = renin angiotensin aldosterone system. SGLT-2 = sodium glucose co-transporter-2. HbA1c = hemoglobin A1c; eGFR = estimated glomerular filtration rate. ACR = albumin-to-creatinine ratio. | |  |

**References**

1 Nederlands Huisartsen Genootschap. NHG-standaard Cardiovasculair Risicomanagement. 2024. https://richtlijnen.nhg.org/standaarden/cardiovasculair-risicomanagement (accessed Nov 9, 2024).

2 Barents ESE, Bilo HJG, Bouma M, *et al.* NHG standaard Diabetes Mellitus Type 2. 2023. https://richtlijnen.nhg.org/standaarden/diabetes-mellitus-type-2 (accessed Nov 9, 2024).

3 American Diabetes Association. 2. Classification and Diagnosis of Diabetes: Standards of Medical Care in Diabetes-2021. *Diabetes Care* 2021; **44**: S15–33.

4 Stevens PE, Ahmed SB, Carrero JJ, *et al.* KDIGO 2024 Clinical Practice Guideline for the Evaluation and Management of Chronic Kidney Disease. *Kidney Int* 2024; **105**: S117–314.
