# Supplementary material for: Effectiveness of a systematic home-based albuminuria screening programme to detect chronic kidney disease in high-risk individuals in primary care (SALINE): a cross-sectional screening study
Source: eClinicalMedicine. 2025 Apr 8;82:103185. doi: 10.1016/j.eclinm.2025.103185 (PMC12005226; doi:10.1016/j.eclinm.2025.103185)
Supplement: Research Protocol [file mmc2.pdf]

Protocol

SALINE study

'Screening for Albuminuria at the first LINE for early identification of chronic kidney disease'

Version 6, 5 January 2023

All rights in this research are reserved by General Practitioners Research Institute B.V. (hereinafter: GPRI) as copyright owner. This research is a copyright protected work within the meaning of article 1 of the Dutch Copyright Act. It is not permitted to reproduce or publish this research, in whole or in part, in any form or by any means, whether electronic, mechanical, by photocopying, recording or any other way, without the prior written permission of GPRI. It is not permitted to independently conduct this research or otherwise independently implement this research, unless a prior written cooperation agreement has been entered into with GPRI. You can contact GPRI to discuss the possibilities of a collaboration.

Alle rechten in dit onderzoek zijn voorbehouden aan GPRI www.gpri.nl

PROTOCOL TITLE

SALINE: 'Screening for Albuminuria at the first LINE for early identification of kidney disease'

**Protocol ID**	GPRI-21004-CKD
**Short title**	**SALINE study**
**Version**	6
**Date**	5 January 2023
**Coordinating investigators**<br>**(commissioned by the sponsor)**	I. van Geer – Postmus, PhD<br>General Practitioners Research Institute<br>Prof. ED Wiersmastraat 5<br>9713 GH Groningen<br>iris.van.geer@gpri.nl
**Principal investigator(s) (in Dutch:**<br>**hoofdonderzoeker/ uitvoerder)**	**Principal**: Prof. Dr. H.J. Lambers Heerspink<br>Department of Clinical Pharmacology<br>University Medical Center Groningen<br>Hanzeplein 1<br>9713 GZ Groningen<br>Tel: 050-3617859<br>E-mail: h.j.lambers.heerspink@umcg.nl<br><br>**Co-principal:** Prof. Dr. R.T. Gansevoort<br>Department of Internal Medicine / Nephrology<br>University Medical Center Groningen<br>Hanzeplein 1<br>9713 GZ Groningen<br>Tel: 050-3616161<br>E-mail: r.t.gansevoort@umcg.nl<br><br>**Co-principal**: Prof. J.W.H. Kocks, MD, PhD<br>General Practitioners Research Institute<br>Prof. ED Wiersmastraat 5<br>9713 GH Groningen<br>Tel: 050-2113898<br>E-mail: janwillem@gpri.nl
**Sponsor (in Dutch:**<br>**verrichter/opdrachtgever)**	General Practiioners Research Institute<br>Prof. ED Wiersmastraat 5<br>9713 GH Groningen
**Subsidising party**	AstraZeneca<br>Prinses Beatrixlaan 582<br>2595 BM Den Haag

**Independent expert**	Dr. C.F.M. Franssen<br>Internist-nephrologist<br>Dept. Nephrology<br>University Medical Center Groningen<br>Hanzeplein 1<br>9713 GZ Groningen<br>The Netherlands
**Laboratory site**	UMCG

PROTOCOL SIGNATURE SHEET

Name	Signature	Date
**Sponsor or legal representative**<br><br>Prof. J.W.H. Kocks<br>General Practitioner<br>Director of General Practitioners Research Institute (GPRI)	DocuSigned by Janwillem Kocks<br>3F5AE5CC28B24FB8B08CA71E12581DD3<br>I approve this document<br>09-jan-2023 | 12:22:45 P	09-Jan-2023
**Principal Investigator**<br><br>Prof. Dr. H.J. Lambers Heerspink<br>Department of Clinical Pharmacology<br>University Medical Center Groningen	DocuSigned by Hiddo Lambers Heerspink<br>Hiddo Lambers Heerspink<br>137B5E96BA974CC1BFC1C7F02B4E3F6B<br>I approve this document<br>10-Jan-2023 | 7:19:49 AM CET	10-Jan-2023
**Coordinating Investigators**<br><br>I. van Geer-Postmus, PhD<br>Senior researcher<br>General Practitioners Research Institute (GPRI)	DocuSigned by Iris van Geer<br>7D7DF45182C047D39CD968923F7E1C37<br>Ik keur dit document goed<br>05-jan-2023 | 1:44:22 PM CET	05-jan-2023

1 Inhoudsopgave

2 INTRODUCTION AND RATIONALE 13

3 OBJECTIVES 16

3.1 Primary objectives 16

3.2 Secondary objectives 16

4 STUDY DESIGN 16

4.1 Phase 1: Targeted screening on albuminuria 17

4.2 Phase 2: Elaborated screening phase 19

4.3 Phase 3: Implementation of care 19

5 STUDY POPULATION 20

5.1 Population (base) 20

5.2 Inclusion criteria 20

5.2.1 Recruitment via pharmacies 20

5.2.2 Recruitment via general practitioners 21

5.3 Exclusion criteria 21

5.4 Sample size calculation 22

6 TREATMENT OF SUBJECTS 22

7 INVESTIGATIONAL PRODUCT 22

8 NON-INVESTIGATIONAL PRODUCT 22

8.1 Name and description of non-investigational product(s) 22

8.1.1 PeeSpot 22

8.1.2 Abbott Affinion™ HbA1c 23

8.1.3 Abbott i-STAT Alinity handheld analyser 23

8.2 Dosages, dosage modifications and method of administration 23

8.3 Preparation and labelling of non Investigational Medicinal Product 23

9 METHODS 24

9.1 Study endpoints 24

9.1.1 Primary endpoints 24

9.1.2 Secondary endpoints 24

9.2 Randomization, blinding and treatment allocation 25

9.3 Study procedures 25

9.4 Withdrawal and replacement of individual subjects 26

9.5 Replacement of individual subjects after withdrawal 26

9.6 Follow-up of subjects withdrawn from treatment ... 27
9.7 Premature termination of the study ... 27
10 SAFETY REPORTING ... 27
10.1 Temporary halt for reasons of subject safety ... 27
10.2 AEs, SAEs and SUSARs ... 27
10.2.1 Adverse events (AEs) ... 27
10.2.2 Serious adverse events (SAEs) ... 27
10.2.3 Follow-up of adverse events ... 28
11 STATISTICAL ANALYSIS ... 28
12 ETHICAL CONSIDERATIONS ... 30
12.1 Regulation statement ... 30
12.2 Recruitment and consent ... 30
12.3 Benefits and risk assessment, group relatedness ... 30
12.4 Compensation for injury ... 30
12.5 Incentives ... 31
13 ADMINISTRATIVE ASPECTS, MONITORING AND PUBLICATION ... 31
13.1 Handling and storage of data and documents ... 31
13.2 Monitoring and Quality Assurance ... 31
13.3 Storage of personal data ... 31
13.4 Storage of collected data ... 32
13.5 Amendments ... 32
13.6 Annual progress report ... 32
13.7 Temporary halt and (prematurely) end of study report ... 32
13.8 Public disclosure and publication policy ... 32
14 STRUCTURED RISK ANALYSIS ... 33
14.1 Potential issues of concern ... 33
15 REFERENCES ... 34
16 APPENDICES ... 37
16.1 Appendix A: Questionnaire ... 37
16.2 Appendix B: Guidelines for cardiovascular risk management (Link to source document) ... 37
16.2.1 Cardiovascular risk profile in men ≥40 and women ≥50 years of age ... 38
16.2.2 Optimal treatment following the NHG guidelines ... 39
16.2.3 SCORE table to estimate risk of cardiovascular mortality in the next 10 years ... 40
16.2.4 NHG Treatment recommendations lipid disorders ... 41

16.2.5 NHG Treatment recommendations hypertension 41

16.3 Appendix C: Medical Adherence Report Scale (MARS) 42

LIST OF ABBREVIATIONS

**ACEi**	ACE-inhibitors
**ARB**	Angiotensin Receptor Blockers
**CKD**	Chronic kidney disease
**CV**	Cardiovascular
**CVD**	Cardiovascular disease
**CVRM**	Cardiovascular risk management
**GDPR**	General Data Protection Regulation
**GP**	General practitioner
**GPRI**	General Practitioners Research Institute
**HbA1c**	Glycosylated hemoglobin
**KDIGO**	Kidney Disease Improving Global Outcomes
**MARS**	Medical Adherence Report Scale
**NHG**	Nederlands Huisartsen Genootschap
**NSAIDS**	Non-steroid anti-inflammatory drugs
**POCT**	Point-of-care test
**RAS**	Renin-angiotensin system
**SGLT2**	Sodium Glucose co Transporter 2
**Sponsor**	The sponsor is the party that commissions the organization or performance of the research, for example a pharmaceutical company, academic hospital, scientific organization or investigator. A party that provides funding for a study but does not commission it is not regarded as the sponsor, but referred to as a subsidizing party.
**UACR**	urinary albumin creatinine ratio
**UAE**	urinary albumin excretion
**WHO**	World Health Organization
**WMO**	Medical Research Involving Human Subjects Act; in Dutch: Wet Medisch-wetenschappelijk Onderzoek met Mensen

SUMMARY

Rationale: Kidney disease, cardiovascular disease and Diabetes Mellitus are closely interrelated. Reducing modifiable cardiovascular risk factors has an effect on preventing both cardiovascular and renal damage. Early detection and appropriate treatment of kidney disease is important as this may prevent future cardiovascular complications and end-organ damage more effectively than intervention in more advanced stages of disease. There is a well-established relationship between albuminuria and renal- and cardiovascular disease. Elevated albuminuria has a relatively high prevalence in the general population (5-9%). The prevalence of albuminuria is even higher in patients with type 2 Diabetes Mellitus (20-40%) and in patients with hypertension (10-15%). Adequate treatment of albuminuria, preferable at early stages can prevent both cardiovascular and renal disease progression. However, scarce epidemiological data show that albuminuria measurements are only conducted in a minority of individuals and disease recognition is suboptimal, even in high-risk groups. This leaves a large proportion of patients not identified, and thus not adequately treated. The current study aims to evaluate if and how early identification of chronic kidney disease by targeted screening of albuminuria levels is feasible in primary care (pharmacies and general practitioners) to optimally discover and treat patients with elevated albuminuria.

Objectives:

Primary objectives:

To perform a study to:

1. Estimate the prevalence of elevated albuminuria in subjects with a high risk of chronic kidney disease in the Netherlands.
2. Evaluate which screening approach would be most effective based on costs per identified patient with elevated albuminuria, via pharmacies or general practices.

Secondary objectives:

1. To estimate the prevalence of unrecognized chronic kidney disease in subjects with a high risk of chronic kidney disease in the Netherlands.
2. To estimate the prevalence of persistent elevated albuminuria under adequate treatment with renin-angiotensin system (RAS) inhibitors in subjects with a high risk of chronic kidney disease in the Netherlands.

3. To assess frequency of cardiovascular risk monitoring by general practitioners after targeted albuminuria screening.
4. To assess changes in cardio-metabolic drug prescriptions after targeted albuminuria screening.

Study design: Cross-sectional study in Dutch pharmacies and general practices.

Study population: Subjects (45-80 years) with a high risk of chronic kidney disease, i.e., having a history or a recent prescription for Diabetes Mellitus, cardiovascular disease, hypertension, lipid disorders or obesity.

Study methods: For this study subjects will be recruited for a targeted screening via pharmacies and general practitioners. For the pharmacies, subjects at risk of albuminuria will be identified as those with a prescription for Diabetes Mellitus, hypertension or lipid disorders. For general practitioners, subjects who have Diabetes Mellitus, cardiovascular disease, hypertension, lipid disorders, or obesity will be selected from electronic medical records. For the targeted screening, subjects are asked to collect urine at home for an albuminuria measurement. In case of elevated albuminuria, subjects will receive a second collection device for a confirmational measurement. If elevated albuminuria is confirmed, subjects will be invited for an elaborated screening visit. Elevated albuminuria will be defined as an urinary albumin creatinine ratio (UACR) ≥3.0 mg/mmol on two occasions in accordance with the prevailing primary care guideline ("NHG Standaard Chronische Nierschade"). If not confirmed, they will receive a third collection device. If the third collection device shows normal albuminuria, the study ends. In case of elevated albuminuria, subjects will be invited for an elaborated screening visit. During this screening visit, cardiovascular risk factors will be assessed, and medication use will be reviewed. A review report following the Dutch primary care guidelines will be provided to the subjects' general practitioner (GP). We aim to examine at least 112 patients with elevated albuminuria at the elaborated screening phase in this study. In the pilot, 57 patients with elevated albuminuria were screened in the elaborate screening phase. These patients were included from 2 pharmacies and 3 general practitioners. For the proposed extension, the pharmacies will be scaled up to a total of 5 and general practitioners to a total of 10. Six months after the study

visit, the frequency of cardiovascular monitoring and treatment changes via medication prescriptions will be monitored via data extraction of the general practitioners and/or pharmacies.

We assume that at least 6300 patients from general practices and pharmacies will be eligible for inclusion in the screening phase.

The costs of both screening approaches per identified patient with elevated albuminuria will be evaluated and compared.

Main study parameters/endpoints:

Primary endpoints

1. Elevated albuminuria, defined as a urinary albumin creatinine ratio (UACR) ≥ 3.0 mg/mmol.
2. Average screening costs per subject that was identified as having elevated albuminuria. All costs considered are mentioned in chapter 9.1.1.

Secondary endpoints

1. Unrecognized chronic kidney disease, defined as elevated albuminuria, not reported by the subject or previously recorded in electronic medical records of the subjects' general practice or pharmacy.
2. Persistent elevated albuminuria, defined as elevated albuminuria persisting despite good adherence (score ≥4 (doses seldom or never missed) reported in MARS) to treatment with RAS-inhibitors for at least 6 weeks prior to elevated albuminuria assessment.
3. Changes in frequency of cardiovascular risk factor monitoring, defined as the difference between the frequency of cardiovascular risk factor monitoring in participants 3 years before and 6 months after targeted albuminuria screening.
4. Changes in cardiometabolic drug prescriptions, defined as changes in any prescription for cardiovascular disease or CKD 6 months after targeted albuminuria screening compared to 3 years before.

Nature and extent of the burden and risks associated with participation, benefit and group relatedness: All selected subjects will be asked to participate on a free-will base and to send a signed informed consent form with a completed short questionnaire to GPRI. They will be

asked to collect urine using the PeeSpot and to send this to GPRI by postal mail for a measurement of albuminuria in the laboratory. Subjects will be informed about the results and, only in case of elevated albuminuria levels, the subject will be asked to repeat the procedure for a confirmational measurement. If the confirmational measurement is negative, a third measurement is needed. If recent (<11 months ago) albuminuria levels were available from the general practice and found to be elevated as well, a second confirmational PeeSpot measurement will not be needed. If recent albuminuria levels recorded by the practice were not elevated, subjects will be considered as not having elevated albuminuria and will be included in the calculation of the prevalences but will not be approached to participate. When albuminuria is confirmed, treatment status will be reviewed, and cardiovascular risk factors will be assessed during a visit. Measurements performed during this visit are non-invasive and part of standard care in general practice and therefore not associated with additional risks. A benefit for the subject could be that elevated albuminuria may be detected earlier because of the screening, and thus comes with health benefits, such as adequate treatment of impaired renal function and medication monitoring by the pharmacy, which has been proven to be effective in preventing adverse events of medication.

Novel aspects

1. It is currently unknown how many subjects are undiagnosed with elevated albuminuria and are thus at a high risk of chronic kidney disease progression.
2. It is unknown which approach to case finding is more (cost) effective in identifying undiagnosed albuminuria in primary care. This study will guide future larger scale programs.
3. It is unknown how many subjects have persistent albuminuria despite adequate treatment with renin-angiotensin system (RAS) inhibitors
4. 4. It is unknown whether the cardiovascular risk monitoring and treatment of subjects change after participation in primary care screening for albuminuria

2 INTRODUCTION AND RATIONALE

Chronic kidney disease (CKD) is a global public health problem with an estimated prevalence that exceeds 10% (1). CKD is associated with an increased risk of cardiovascular events, death and high healthcare costs (3). The absolute CKD burden is likely to rise as the prevalence of the main drivers for CKD, Type 2 Diabetes Mellitus and hypertension, continue to increase driven by the obesity epidemic and the aging population (2–4).

There is a remarkable overlap between the kidney and the heart regarding risk factors for disease progression (5). Classic cardiovascular (CV) risk profiling is based on age, gender, blood pressure, cholesterol, smoking, body mass index and type 2 diabetes (5). Reducing modifiable CV risk factors has an effect on preventing both CV and renal damage. Despite available cardiovascular risk management programs in general practice, CV and renal protection remains suboptimal, in that many patients progress to end stage renal disease or show CV morbidity or mortality despite guideline-based treatment (5). Thus, there is a need for early identification and preventive measures on the one hand and new therapies on the other hand.

An increase in urinary albumin excretion (UAE) has been shown to be an important indicator of renal function and cardiovascular disease progression, independent of widely known cardiovascular risk factors. Measurement of albuminuria has therefore been included in Dutch cardiovascular risk management programs applied in general practice (6,7). The Prevention of Renal and Vascular End stage Disease (PREVEND) study investigated the natural course of UAE and its relationship with renal and cardiovascular disease among inhabitants of the city of Groningen, aged 28-75 years. In a cohort of responders with a urinary albumin concentration >10 mg/L (n = 6,000 out of 40,856) and a randomly selected control group of 2,592 persons, they found that albuminuria progressed to a higher class in 11% during a median follow-up of 4 years and this risk increased with an increasing baseline UAE (6). UAE remained stable in 79% of subjects and regressed in 10% of subjects.

Albuminuria is generally defined by a urinary albumin-to-creatinine ratio (UACR) and is considered to be an early marker of glomerular kidney disease (8). During the last three

decades it has been shown that elevated albuminuria has a relatively high prevalence in patients with type 2 diabetes (20 – 40%), hypertension (15 – 25%) and the general population (5 – 9%) (2,3,9). As a result, the World Health Organization (WHO) has recently accepted elevated albuminuria as part of the definition of CKD (10). Current guidelines recognize albuminuria as central to CKD staging and provide criteria for referral and treatment (11). The prevalence of albuminuria in Dutch primary care subjects at risk of developing chronic kidney disease per risk group is currently unknown.

A progressive yearly deterioration of renal function is frequent in patients with diabetes and hypertension that results in elevated albuminuria (12). ACE-inhibitors (ACEi) and Angiotensin Receptor Blockers (ARBs) are recommended by clinical practice guidelines to lower albuminuria and blood pressure in patients with hypertension and diabetes (11). Despite the use of current therapies, the risk of developing CKD remains high (2). ACEi and ARBs reduce albuminuria by approximately 30%, leaving albuminuria not optimally controlled in many patients. To further optimize cardiovascular and kidney prognosis, Sodium Glucose co Transporter 2 (SGLT2) inhibitors, such as dapagliflozin, are a promising new class of therapy. SGLT2 inhibitors have been demonstrated to lower albuminuria and protect the kidney in patients with established chronic kidney disease (CKD) with or without diabetes (13). Whether this effect can be extended to a broader population of individuals at early stages of chronic kidney disease is currently tested in a randomized clinical trial.

Although albuminuria tests have been proven successful in identifying high-risk CKD patients, previous studies have shown that albuminuria measurements are only conducted in a minority of individuals at risk, even in high-risk groups (9). A study in England evaluated the prevalence of both recognized and unrecognized CKD in a study conducted at pharmacies that selected patients with CKD risk factors (Diabetes Mellitus, cardiovascular disease, history of CKD, hypertension, smoking). They reported that 113 of 720 (16%) patients with CKD risk factors had unrecognized CKD (14). A similar study in the United States found that among patients with type 2 Diabetes Mellitus more than half of the patients showed changes in renal markers indicating renal damage. However, only 12% of them had CKD documented in their chart review (4).

It is currently unknown how many primary care patients are at an unrecognized early stage of chronic kidney disease and who many of them are undertreated in the Netherlands.

Unlike other chronic diseases that have established screening strategies, no consensus on the early identification and intervention for CKD is reached by governments and health systems. Guidelines to manage and evaluate early CKD are available but not universally adopted (15). The 2019 international conference from the Kidney Disease Improving Global Outcomes (KDIGO) with clinicians and scientists delivered guidelines for CKD screening. The group concluded that efforts for early detection of CKD should first be implemented among people with hypertension, Diabetes Mellitus and cardiovascular disease.
General practitioners and pharmacists are frontline health care professionals who play an important role in early identification of patients at risk of CKD. Cardiovascular risk management programs are developed by multidisciplinary teams in the Netherlands that provide recommendations on how to estimate the patient’s cardiovascular risk profile (7). Cardiovascular risk assessment, including albuminuria testing should be part of routine care in the Netherlands as stated in the Dutch guidelines. However, only a minority of patients were found to be tested on albuminuria in general practice (16).

A public health benefit of identifying subjects with mild to moderate CKD in the early stages is suggested. Insights in the prevalence of elevated albuminuria, the proportion unrecognized chronic kidney disease and the proportion of high-risk subjects with optimal treatment are essential to evaluate the (cost-) effectiveness of a targeted screening in primary care. Therefore, the aim of the current study is to evaluate if and how early identification of CKD by targeted screening using albuminuria-levels is feasible in primary care, to optimally discover and treat subjects with elevated albuminuria.

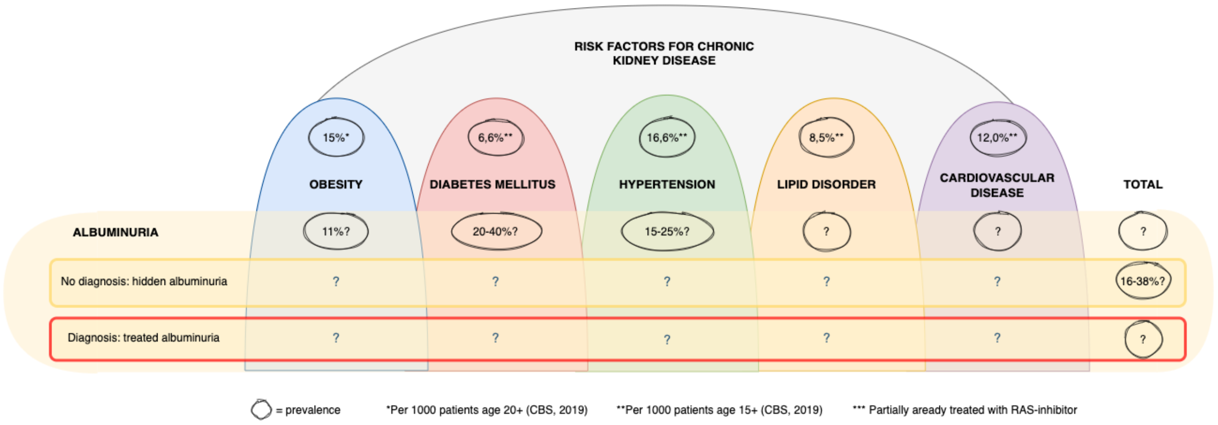

Figure 1. Prevalences of different risk factors for chronic kidney disease (obesity, Diabetes Mellitus, hypertension, lipid disorders and cardiovascular disease) reported in the Dutch population with reported and unknown prevalences of elevated albuminuria in subgroups of patients with (combinations of) risk factors.

3 OBJECTIVES

3.1 Primary objectives

To perform a study to:

1. Estimate the prevalence of elevated albuminuria in subjects with a high risk of chronic kidney disease in the Netherlands.
2. Evaluate which screening approach would be most effective based on costs per identified patient with elevated albuminuria, via pharmacies or general practices.

3.2 Secondary objectives

1. To estimate the prevalence of unrecognized chronic kidney disease in subjects with a high risk of chronic kidney disease in the Netherlands.
2. To estimate the prevalence of persistent elevated albuminuria under adequate treatment with renin-angiotensin system (RAS) inhibitors in subjects with a high risk of chronic kidney disease in the Netherlands
3. To assess frequency of cardiovascular risk monitoring by general practitioners after targeted albuminuria screening.
4. To assess changes in cardio-metabolic drug prescriptions after targeted screening.

4 STUDY DESIGN

The SALINE study is a cross-sectional study that consists of two phases: *a targeted screening phase*, and (only if elevated albuminuria is confirmed) an *elaborated screening phase*. An overview of the study design is shown in Figure 2.

Potential participants will be recruited via:

1. Pharmacies through database selection on prescribed medication for Diabetes Mellitus, hypertension or lipid disorders.
2. General practices through a history of Diabetes Mellitus, hypertension, cardiovascular disease, lipid disorders and/or obesity in patient electronic medical records using VIPlive.

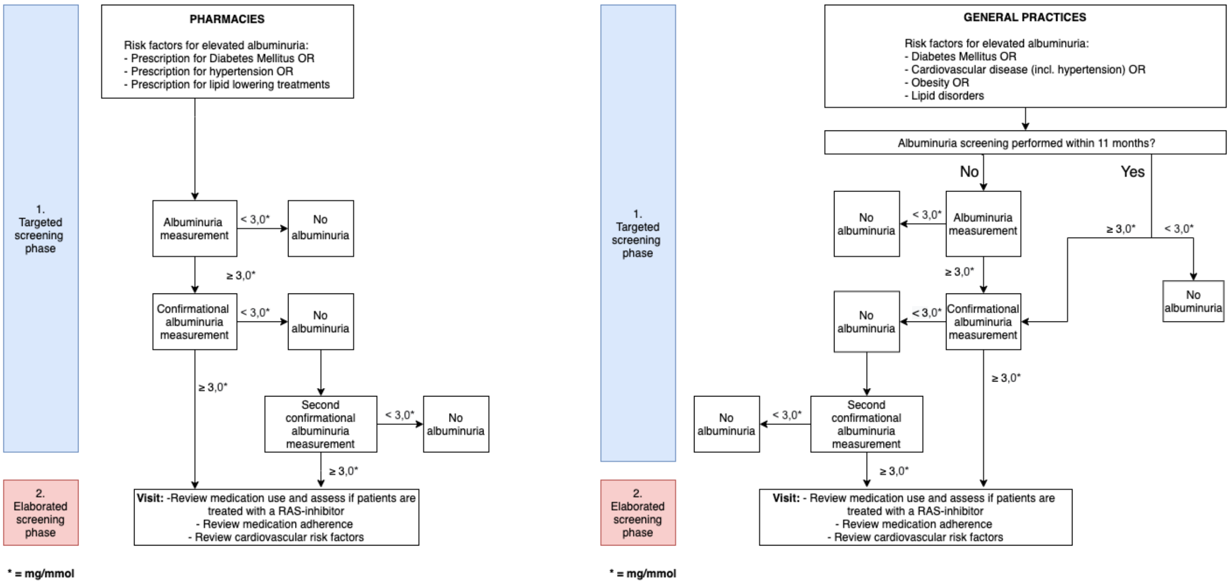

Figure 2. Overview of the study design

In the pilot study, 2 pharmacies and 3 general practitioners were included. For the extension, 3 pharmacies and 7 general practitioners will be recruited.

We assume that at least 6300 patients from general practices and pharmacies will be eligible for inclusion in the screening phase. We aim to examine at least 112 patients with elevated albuminuria at the elaborated screening phase in this study. In the pilot study, 57 patients were examined at the elaborated screening phase.

The effectiveness of both screening approaches in finding elevated albuminuria will be evaluated by calculating the total screening costs per identified patient with elevated albuminuria and compared. An amendment to the protocol may be proposed for ethical approval to extent screening to a larger population based on the results.

4.1 Phase 1: Targeted screening on albuminuria

Targeted screening via pharmacies

Pharmacies will select eligible subjects to be invited for albuminuria screening from their database, based on delivered medication that has an indication for hypertension, Diabetes Mellitus, or lipid disorders in the last 6 months, based on combined information on ATC codes (chapter 4.2.1) for prescribed medication and available registered information on subjects having these indications.

Targeted screening via general practitioners

General practitioners will select eligible subjects for albuminuria screening from subjects' electronic medical records, based on the presence of risk factors for elevated chronic kidney disease. Using a VIPlive selection report, subjects will be selected based on recording of one or more of the following:

3. An ICPC code for Diabetes Mellitus
4. An ICPC code for cardiovascular disease, including hypertension
5. An ICPC code for lipid disorders
6. An ICPC code for obesity or a calculated BMI ≥30 kg/m^2, based on height and weight

If albuminuria status was recently (within 18 months) assessed by the general practice and was found to be 'normal' (< 3.0 mg/mmol), subjects will not be invited for albuminuria screening. All other subjects with an increased risk of chronic kidney disease will be invited by their general practice to participate.

Potential overlap

Potential overlap in subject recruitment occurring when participating pharmacies are providing services to subjects of also participating general practitioners will be avoided as much as possible. In case of known overlap at the recruitment phase, data available from the general practitioner will be preferred. All subjects being registered as a patient from a participating GP in the pharmacy database will be excluded from the selection list from the pharmacy.

Albuminuria screening using PeeSpot

Selected subjects will receive an invitation to participate in the screening survey from their pharmacy or general practice by postal mail, including an informational letter about the study, an informed consent form, a short questionnaire to be completed (Appendix 16.1)

and a urine collection device (PeeSpot) with instructions. The participant will be asked to send the signed informed consent form and completed questionnaire and the collected urine to the General Practitioners Research Institute (GPRI) by postal mail (without costs). If the laboratory measures 'normal' albuminuria (<3.0 mg/mmol), GPRI will inform the subject. In case of elevated albuminuria (≥3.0 mg/mmol), GPRI will be informed to send a second PeeSpot for a confirmational measurement if informed consent has been received. If this second measurement is also elevated (≥3.0 mg/mmol), subjects will be invited for an elaborated screening visit performed by GPRI (phase 2). A second confirmational measurement of elevated albuminuria will not be required when a recent (within 11 months) measurement performed by the general practice also indicated elevated albuminuria.

4.2 Phase 2: Elaborated screening phase

When there is no elevated albuminuria at phase 1, a letter with the results will be sent to the participant, and no further actions from the participant are required. Subjects will be carefully informed about the results.

When elevated albuminuria is confirmed (≥3.0 mg/mmol on two occasions), subjects are invited for an elaborated screening visit with a researcher. At the start of the visit, participants will be asked to provide informed consent. Next, CKD/CVD risk factors will be assessed and the participant's medication use, and medication adherence will be reviewed during an interview. GPRI will produce a report of their findings which will be shared with the general practice, including recommendations for additional measurements or treatment changes if applicable.

4.3 Phase 3: Implementation of care

In phase 3 of the SALINE study we will determine if cardiovascular risk factor monitoring and cardiometabolic drug prescriptions changes within 6 months after participants have participated in the screening phases of the study. To this end, data about cardio-metabolic risk factor monitoring and prescriptions will be extracted from the database of the general practitioner and/or pharmacies involving all patients who qualified for the SALINE study (thus both those who participate in SALINE and those who did not). We will then determine

the frequency of cardio-metabolic risk factor monitoring and if drug prescriptions have occurred in the 6 months following the albuminuria targeted screening and compare those with particpiants who did not participate in SALINE.

5 STUDY POPULATION

5.1 Population (base)

Subjects with a high risk of (developing) chronic kidney disease will be recruited in primary care practices and pharmacies from the General Practitioners Research Institute (GPRI) network. The organizational model of GPRI is based on the participation of independent practices and pharmacies in the Netherlands, well-trained GPs, pharmacists and their staff. Male and female subjects of 45-80 years of age that meet the inclusion criteria will be eligible for the study.

5.2 Inclusion criteria

5.2.1 Recruitment via pharmacies

- Age of 45-80 years
- Delivered medication within the last 6 months for one of the following indications (available recorded information on indication will be considered):

Indication	Medication	ATC code
Diabetes Mellitus	Glucose lowering drugs, excluding insulins	A10B*
	Other drugs used in diabetes, including aldose reductase inhibitors<br>Insulin and analogues	A10X*<br><br>A10A*
Hypertension	ACE-inhibitors<br>Angiotensin receptor blocker (ARB)<br>Diuretics<br>Calcium-antagonists<br>Betablockers, excluding propranolol	C09A, C09B<br>C09C, C09D<br>C03<br>C08<br>C07*, excluding C07AA05
Lipid lowering treatments	HMG CoA reductase inhibitors (statins)<br><br>Ezetimibe	C10AA, C10BA, C10BX<br>C10AX09

5.2.2 Recruitment via general practitioners

- 45-80 years old
- At least one of the following indications (based on ICPC codes and recorded laboratory measurements (e.g., blood pressure).

Indication	ICPC code
○ Diabetes Mellitus	T90.01 & T90.02
○ Obesity (BMI ≥ 30 kg/m2)	T82
○ Lipid disorders	T93.0*
○ Hypertension	K86 (without organ damage)<br>K87 (with organ damage)
○ Established cardiovascular disease (see indications in rows below)	
1. Acute coronary syndrome	M80
2. Angina Pectoris	K74
3. Coronary revascularization/coronary sclerosis	Surgery
4. Transient Ischemic Attack (TIA) or stroke	K89, K90
5. Aorta aneurysm	K99.01
6. Claudicatio intermittens	K92.01
7. Peripheral revascularization	Surgery
8. (Chronic) ischemic heart disease	K76
9. Myocardial infarction	K75
10. Atherosclerosis	K91
11. Decompensatio cordis	K77

5.3 Exclusion criteria

- Inability to understand and sign the informed consent form
- Pregnancy
- Know normal (<3mg/mmol) albuminuria status with 18 months prior

5.4 Sample size calculation

We aim to examine at least 112 patients with elevated albuminuria at the elaborated screening phase in this study. In the pilot study, 57 patients were examined at the elaborated screening phase. In the pilot study, 2 pharmacies and 3 general practitioners were included. For the extension, 3 pharmacies and 7 general practitioners will be recruited.We assume that at least 6300 patients from general practices and pharmacies will be eligible for inclusion in the screening phase.

6 TREATMENT OF SUBJECTS

Not applicable as there are no direct treatment interventions in this study. This is a targeted screening study, in which subjects with an increased risk of elevated albuminuria are identified and screened for albuminuria. If albuminuria is elevated, the CKD/CVD risk factors are assessed, and medication will be reviewed during an elaborated screening visit following standard guidelines. These results will be shared with the subjects' general practitioner.

7 INVESTIGATIONAL PRODUCT

Not applicable

8 NON-INVESTIGATIONAL PRODUCT

8.1 Name and description of non-investigational product(s)

8.1.1 PeeSpot

Albuminuria will be measured in a laboratory as the urinary albumin-to-creatinine ratio (UACR) using the PeeSpot method. Elevated albuminuria will be defined as a urinary albumin creatinine ratio (UACR) ≥3.0 mg/mmol (equivalent to ~30mg/g). A confirmatory test using the same methods will be performed in case of elevated albuminuria, because of intra-individual and biological variation, following the Clinical Practice Guideline for the Evaluation and Management of Chronic Kidney Disease (10), except when elevated albuminuria was also previously assessed within 11 months prior to the PeeSpot measurement.

For the PeeSpot measurement, subjects will receive a urine collection device that consists of a tube with an inert absorption felt which is clamped in a holder with handle (17). The absorption felt has the property to absorb urine well and quickly. After centrifugation all

substances are obtained again from the felt with 100% recovery (17,18). The utility of the PeeSpot is tested in multiple studies, where the PeeSpot was found to be reliable and suitable for chemical analyses of urine samples (17). In order to use the PeeSpot, subjects need to open the tube and take the PeeSpot out. Subjects need to urinate over the absorption felt for about five seconds and place the PeeSpot back in a leakproof tube and firmly close the lid. The PeeSpot can be sent in a 'medical package' envelope to the laboratory. The tube is centrifuged in the laboratory and the urine obtained is suitable for all biochemical tests. Subjects find the PeeSpot easy to use (17).

8.1.2 Abbott Affinion™ HbA1c

During the screening visit, where CKD/CVD risk factors will be assessed, HbA1c will be measured with a point-of-care-test (Afinion™ HbA1c). The HbA1c-level of the subject is measured within three minutes using a couple drops of (capillary) blood of the subject.

8.1.3 Abbott i-STAT Alinity handheld analyser

Creatinine will be measured with a point of care i-STAT Alinity handheld analyser (Abbott). This system operates with the i-STAT Alinity Creatinine (CREA) cartridges. A drop of blood is applied on the cartridge, followed by insertion in the machine. This machine provides current creatinine measurements in two minutes.

8.2 Dosages, dosage modifications and method of administration

Not applicable.

8.3 Preparation and labelling of non Investigational Medicinal Product

Not applicable.

9 METHODS

9.1 Study endpoints

9.1.1 Primary endpoints

1. Elevated albuminuria, defined as a urinary albumin creatinine ratio (UACR) ≥ 3.0 mg/mmol.
2. Average screening costs per subject that was identified as having kidney disease by screening for albuminuria. All costs considered are mentioned below (Table 1)

Table 1. Costs associated with screening

Costs of the PeeSpot
Laboratory costs
Printing and postal mailing costs of all invitations and needed information
Labor costs needed to recruit and start GP's and pharmacies
Labor costs of training GP's and pharmacies
Costs of extracting patients eligible for inclusion

9.1.2 Secondary endpoints

1. Unrecognized chronic kidney disease, defined as elevated albuminuria, not reported by the subject and not previously recorded in electronic medical records of the subjects' general practice or pharmacy.
2. Persistent elevated albuminuria, defined as elevated albuminuria persisting despite good adherence to treatment with RAS-inhibitors for at least 6 weeks prior to elevated albuminuria assessment (score ≥4 on the Medical Adherence Report Scale (MARS)) (Appendix C, paragraph 16.3).
3. Change in frequency of cardiovascular risk factor monitoring, defined as the difference between the frequency of cardiovascular risk factor monitoring in participants 3 years before and 6 months after targeted albuminuria screening.
4. Changes in cardiometabolic drug prescriptions, defined as changes in any prescription for cardiovascular disease or CKD 6 months after targeted albuminuria screening compared to three years before.

9.2 Randomization, blinding and treatment allocation

Not applicable

9.3 Study procedures

The targeted screening will be performed in the pharmacies and general practices that included the subjects. The visit and cardiovascular risk factor measurements will be performed by GPRI or the University Medical Center Groningen. Albuminuria measurements will be performed by the University Medical Center Groningen.

Targeted screening phase

Pharmacies and GPs select potential subjects based on the medical history and last 6 months of prescriptions and registered indications, as described in section 5.2. During this phase subjects will be asked to give written informed consent to participate in the study. Subjects will collect urine samples using the PeeSpot and sent the samples to the laboratory. Furthermore, they receive a questionnaire to collect subject characteristics. The informed consent form and the subject characteristics questionnaire are sent to the General Practitioners Research Institute (GPRI). GPRI will receive the results of the urine tests. If albuminuria is elevated, subjects receive a second PeeSpot to perform a confirmational measurement. If subjects are recruited via GPs and have an elevated albuminuria measurement performed less than 11 months ago, only one extra albuminuria measurement is necessary.

Elaborated screening phase

In case of elevated albuminuria, subjects will be invited to attend an elaborated screening visit with a researcher of GPRI at the pharmacy, GP practice or other convenient location near the subject.

The following data will be collected by interview:

1. Medical history of
    a. Kidney disease
    b. Albuminuria
    c. Diabetes Mellitus, type 1 or 2, including a history of keto-acidosis

d. Cardiovascular disease

e. Hypertension

f. Lipid disorders

2. Medication use over the last 6 weeks
3. Medication adherence measured with the medication adherence report scale-5 (MARS-5) (appendix 16.3)
4. Smoking status, never, current, or ex-smoker

The following data will be collected by measurement:

1. Pulse rate
2. Systolic and diastolic tension (mean of 3 measurements)
3. Height
4. Weight (kg)
5. Creatinine and HbA1c in capillary blood

A report will be sent to the general practitioner based on these findings. The general practitioner will be advised to follow the NHG guidelines for cardiovascular management (Appendix 0) and to adjust treatment, if necessary.

Implementation of care

After the study visits have been completed, data extractions from the general practitioners and/or pharmacies will be performed to retrieve data of cardiovascular risk monitoring and changes in cardiometabolic medication prescriptions from 6 months after the SALINE study visit of the participant.

9.4 Withdrawal and replacement of individual subjects

Subjects can leave the study at any time for any reason if they wish to do so without any consequences. The investigator can decide to withdraw a subject from the study. There are no specific criteria for withdrawal. Every subject that meets the inclusion criteria is invited, therefore replacement is not possible.

9.5 Replacement of individual subjects after withdrawal

Not applicable

9.6 Follow-up of subjects withdrawn from treatment

Not applicable

9.7 Premature termination of the study

The end of the study is defined as the end of the elaborated screening visit phase. There are no predefined criteria for premature termination of the study. If, however, the investigator/sponsor terminates the study without prior agreement of the subsidizing party, the investigator/sponsor should instantly inform the subsidizing party and provide them with a detailed written explanation for the termination of the study. If the subsidizing party terminates the trial, the subsidizing party should instantly inform the investigator/sponsor with a detailed explanation for the termination of the study.

10 SAFETY REPORTING

10.1 Temporary halt for reasons of subject safety

In accordance with section 10, subsection 4, of the WMO, the sponsor will suspend the study if there is sufficient ground that continuation of the study will jeopardize subject health or safety. The sponsor will notify the accredited METC without undue delay of a temporary halt including the reason for such an action. The study will be suspended pending a further positive decision by the accredited METC. The investigator will take care that all subjects are kept informed.

10.2 AEs, SAEs and SUSARs

10.2.1 Adverse events (AEs)

Adverse events are defined as any undesirable experience occurring to a subject during the study. All adverse events reported spontaneously by the subject or observed by the investigator or his staff during the elaborate screening phase will be recorded.

10.2.2 Serious adverse events (SAEs)

A serious adverse event is any untoward medical occurrence or effect that

- Results in death;
- Is life threatening (at the time of the event);
- Requires hospitalisation or prolongation of existing in subjects’ hospitalisation;
- Results in persistent or significant disability or incapacity;

- Any other important medical event that did not result in any of the outcomes listed above due to medical or surgical intervention but could have been based upon appropriate judgement by the investigator.

An elective hospital admission will not be considered as a serious adverse event.
Any SAE that happens during the elaborate screening phase will be reported
The sponsor will report the SAEs through the web portal *ToetsingOnline* to the accredited METC that approved the protocol, within 7 days of first knowledge for SAEs that result in death or are life threatening followed by a period of maximum of 8 days to complete the initial preliminary report. All other SAEs will be reported within a period of maximum 15 days after the sponsor has first knowledge of the serious adverse events.

10.2.3 Follow-up of adverse events

All AEs will be followed until they have abated, or until a stable situation has been reached. Depending on the event, follow up may require additional tests or medical procedures as indicated, and/or referral to the general physician or a medical specialist.
SAEs need to be reported till end of study within the Netherlands, as defined in the protocol.

11 STATISTICAL ANALYSIS

Characteristics of subjects recruited will be described as mean values with standard deviation or median with interquartile range for continuous variables. Categorical variables will be described as numbers and percentages of the subjects per category.
The overall prevalence of elevated albuminuria, and prevalences of unrecognized and persistent elevated albuminuria despite a RAS-inhibitor are described as percentages with 95% confidence intervals. The number of eligible patients from general practices who fulfilled the inclusion criteria but had an albuminuria level <3 mg/mmol recorded within 18months prior to selection (and where therefore not invited to participate), will be included in the denominator to calculate the prevalences.
To compare effectiveness and costs of screening on elevated albuminuria in pharmacies and in GP-care, the costs for the targeted screening strategies in both pharmacies and GPs will be calculated.
For each approach, all costs associated with the screening will be added and divided by the total number of subjects identified with confirmed elevated albuminuria. A screening-

approach will be considered more effective than the other if the costs per identified patient are ≥10% lower.

Frequencies of cardiovascular monitoring and treatment changes are summarized as numbers and percentages with 95% confidence intervals.

Evaluating non-response:

Aggregated data of characteristics from the total population of subjects that is eligible for screening will be collected from each general practice through VIPLive and will be combined to describe pooled characteristics of the total eligible population from general practices (Table 2).

Table 2. Distribution of characteristics of the study population selected from participating general practices

Characteristic	Distribution
Age	Mean (standard deviation)
Sex, female	Number (%)
Diabetes Mellitus	Number (%)
Elevated albuminuria in last 11 months	Number (%)
Lipid disorder	Number (%)
Obesity	Number (%)
Hypertension	Number (%)
Cardiovascular disease	Number (%)
Kidney disease	Number (%)

Pharmacies will be asked to provide similar data used to select their subjects.

The distribution of subject characteristics in the total selected population will be compared with the distribution among responders using collected information to evaluate occurrence of systematic response bias. Collected information of responders will be used for post-stratification and weighting methods to account for potential response bias in the estimation of prevalences (19).

12 ETHICAL CONSIDERATIONS

12.1 Regulation statement

This study will be conducted in accordance with the principles of the declaration of Helsinki (version October 2013) and in accordance with the Medical Research Involving Human Subjects Act (WMO).

12.2 Recruitment and consent

General practices and pharmacies from the network of GPRI in the Netherlands will be directly approached to participate in the study and via other channels, like social media and newsletters. If a pharmacy or general practice is interested in participation, more information regarding the study will be provided. Subsequently, an agreement will be signed between GPRI and the participating party. In the participating practices and pharmacies subjects will be recruited by targeted screening. It is on the discretion of the recruiting GP or pharmacist if he/she deems the subject eligible for participation based on the inclusion- and exclusion criteria mentioned in chapter 4.2 and 4.3. Subjects are informed about the study in writing with explanation of the study, advantages and disadvantages of participating and contact information of the research team members working on the study. If the subject is interested in participation, the subject will be asked to sign the informed consent form. Subjects will not be financially compensated for participation.

12.3 Benefits and risk assessment, group relatedness

Participation in the study is on a free-will base. Screening measurements are non-invasive and therefore no risks are associated with participation. The point of care measurements of HbA1c and creatinine are performed using a minimal invasive finger prick test. An indirect benefit could be that elevated albuminuria could be detected earlier because of the screening, and thus comes with health benefits. Another indirect benefit is that the medication is checked and compared to the most recent GP guidelines and on the GPs discretion be optimized if needed.

12.4 Compensation for injury

The sponsor/investigator has a liability insurance which is in accordance with article 7 of the WMO.

The sponsor (also) has an insurance which is in accordance with the legal requirements in the Netherlands (Article 7 WMO). This insurance provides cover for damage to research subjects through injury or death caused by the study.
The insurance applies to the damage that becomes apparent during the study or within 4 years after the end of the study.

12.5 Incentives

Participation of subjects in the study is a free-will decision. Subjects do not need to pay for the urine tests or study assessments. Travel expenses will be reimbursed.
Participating pharmacies and GPs will be reimbursed in accordance to the time-investments they will make.

13 ADMINISTRATIVE ASPECTS, MONITORING AND PUBLICATION

13.1 Handling and storage of data and documents

13.2 Monitoring and Quality Assurance

Monitoring will be done following a monitoring plan.

13.3 Storage of personal data

The handling of personal data will proceed confidentially. The pharmacy and general practitioner are offered the opportunity to use an ISO-certified post order company specialized in security for sending out the invitations. The data of eligible subjects will be handled in the secure and specialized environment of the post order company and will be destroyed after 21 days. Subjects receive a unique study identification number. GPRI has no access to the data of eligible subjects who will be invited. Only when subjects have signed the informed consent and have provided data by returning the completed questionnaire, GPRI receives pseudonymized patient data because the IC and questionnaire are marked by the identification number. During the analysis phase, all data provided by patients that signed the informed consent form are used pseudonymized. The identification number is not linked to the participant's personal information. A subject identification code list will be used to link the data to a participant, the key to the code will be safeguarded by the coordinating investigator. The de-identification process will only be used to contact the participant or his/her general practitioner if they have given consent.

13.4 Storage of collected data

The source documents, or informed consents and investigator files will be archived for 25 years. Data handling and storage complies with the General Data Protection Regulation (GDPR).

13.5 Amendments

Amendments are changes made to the research after a favourable opinion by the accredited METC has been given. All amendments will be notified to the METC that gave a favourable opinion.

13.6 Annual progress report

The sponsor/investigator will submit a summary of the progress of the trial to the accredited METC once a year. Information will be provided on the date of inclusion of the first subject, numbers of subjects included and numbers of subjects that have completed the trial, serious adverse events/ serious adverse reactions, other problems, and amendments.

13.7 Temporary halt and (prematurely) end of study report

The investigator/sponsor will notify the accredited METC of the end of the study within a period of 8 weeks. The end of the study is defined as the subjects' last visit.

The sponsor will notify the METC immediately of a temporary halt of the study, including the reason of such an action.

In case the study is ended prematurely, the sponsor will notify the accredited METC within 15 days, including the reasons for the premature termination.

Within one year after the end of the study, the investigator/sponsor will submit a final study report with the results of the study, including any publications/abstracts of the study, to the accredited METC.

13.8 Public disclosure and publication policy

Findings of the study will be published in peer-reviewed scientific journals. Additionally, we aim to present the results as abstracts at (inter)national conferences. In our publications, the identity of the subjects will not be disclosed in any way.

14 STRUCTURED RISK ANALYSIS

14.1 Potential issues of concern

Given the nature of the PeeSpot device and the minimal invasive finger prick test no potential issues of concern regarding risk are foreseen.

15 REFERENCES

1. Vassalotti JA, Centor R, Turner BJ, Greer RC, Choi M, Sequist TD. Practical Approach to Detection and Management of Chronic Kidney Disease for the Primary Care Clinician. American Journal of Medicine [Internet]. 2016;129(2):153-162.e7. Available from: http://dx.doi.org/10.1016/j.amjmed.2015.08.025
2. Bramlage P, Lanzinger S, van Mark G, Hess E, Fahrner S, Heyer CHJ, et al. Patient and disease characteristics of type-2 diabetes patients with or without chronic kidney disease: An analysis of the German DPV and DIVE databases. Cardiovascular Diabetology [Internet]. 2019;18(1):1–12. Available from: https://doi.org/10.1186/s12933-019-0837-x
3. Hounkpatin HO, Harris S, Fraser SDS, Day J, Mindell JS, Taal MW, et al. Prevalence of chronic kidney disease in adults in England: Comparison of nationally representative cross-sectional surveys from 2003 to 2016. BMJ Open. 2020;10(8).
4. Szczech LA, Stewart RC, Su HL, DeLoskey RJ, Astor BC, Fox CH, et al. Primary care detection of chronic kidney disease in adults with Type-2 diabetes: The ADD-CKD study (awareness, detection and drug therapy in type 2 diabetes and chronic kidney disease). PLoS ONE. 2014;9(11):1–16.
5. L S, N H, PE de J, D de Z, SJ B, RT G. Predictors of progression in albuminuria in the general population: results from the PREVEND cohort. PloS one [Internet]. 2013 May 27 [cited 2021 Jul 19];8(5). Available from: https://pubmed.ncbi.nlm.nih.gov/23723966/
6. Brantsma AH, Atthobari J, Bakker SJL, de Zeeuw D, de Jong PE, Gansevoort RT. What predicts progression and regression of urinary albumin excretion in the nondiabetic population? Journal of the American Society of Nephrology. 2007 Feb;18(2):637–45.
7. NHG. Cardiovasculair risicomanagement | NHG-Richtlijnen [Internet]. [cited 2021 Jul 13]. Available from: https://richtlijnen.nhg.org/standaarden/cardiovasculair-risicomanagement
8. Saydah, Sharon H., Pavkov,, Meda E. , Zhang, Cindy., Lacher, David A., Eberhardt, Mark S., Rios Burrows, Nilka., Narva, Andrew S., Eggers, Paul W., and Williams DE. Albuminuria Prevalence in First Morning Void Compared with Previous Random Urine

from Adults in the National Health and Nutrition Examination Survey, 2009–2010. Clinical Chemistry. 2013;59(4):675–83.

9. Gasparini A, Evans M, Coresh J, Grams ME, Norin O, Qureshi AR, et al. Prevalence and recognition of chronic kidney disease in Stockholm healthcare. Nephrology Dialysis Transplantation. 2016;31(12):2086–94.

10. KDIGO Board Members. Kidney disease: KDIGO 2012 clinical practice guideline for the evaluation and management of chronic kidney disease.

11. NHG. Chronische nierschade | NHG-Richtlijnen [Internet]. [cited 2021 Jul 13]. Available from: https://richtlijnen.nhg.org/standaarden/chronische-nierschade#samenvatting-stroomdiagram

12. Polonia J, Azevedo A, Monte M, Silva JA, Bertoquini S. Annual deterioration of renal function in hypertensive patients with and without diabetes. Vascular Health and Risk Management. 2017;13:231–7.

13. DC W, BV S, N J, GM C, T G, FF H, et al. Effects of dapagliflozin on major adverse kidney and cardiovascular events in patients with diabetic and non-diabetic chronic kidney disease: a prespecified analysis from the DAPA-CKD trial. The lancet Diabetes & endocrinology [Internet]. 2021 Jan 1 [cited 2021 Jul 20];9(1):22–31. Available from: https://pubmed.ncbi.nlm.nih.gov/33338413/

14. al Hamarneh YN, Hemmelgarn B, Curtis C, Balint C, Jones CA, Tsuyuki RT. Community pharmacist targeted screening for chronic kidney disease. Canadian Pharmacists Journal. 2016;149(1):13–7.

15. Shlipak MG, Tummalapalli SL, Boulware LE, Grams ME, Ix JH, Jha V, et al. The case for early identification and intervention of chronic kidney disease: conclusions from a Kidney Disease: Improving Global Outcomes (KDIGO) Controversies Conference. Kidney International. 2021;99(1):34–47.

16. Leemrijse C Chantal J, Korevaar J Johanna C, Nederlands instituut voor onderzoek van de gezondheidszorg (Utrecht). Vroegopsporing van mensen met een verhoogd risico op hart- en vaatziekten. 2019;

17. Hessels J, Cairo DW, Slettenhaar M, Dogger M. PeeSpot; urine home collection device Innovatieve methode voor verzamelen van portie urine. Nederlands Tijdschrift voor Klinische Chemie en Laboratoriumgeneeskunde. 2011;36(4):249–51.

18. HESSELS+GROB bv Evaluatie van PeeSpot ® urine colection device. 2011 [cited 2021 Jul 23]; Available from: www.hesselsgrob.com

19. TC C, J C, MK R, LK M, THI F. National Health and Nutrition Examination Survey, 2015-2018: Sample Design and Estimation Procedures. Vital and health statistics Series 2, Data evaluation and methods research [Internet]. 2020 Apr 1 [cited 2021 Jul 23];(184):1–35. Available from: https://pubmed.ncbi.nlm.nih.gov/33663649/

16 APPENDICES

16.1 Appendix A: Questionnaire

Vragenlijst SALINE-onderzoek

Datum ingevuld: ☐☐ - ☐☐ - ☐☐☐☐

Wat is uw adres:	
Wat is uw telefoonnummer:	
Wat is uw e-mailadres:	
Wat is uw leeftijd?	
Wat is uw geslacht?	○ Man<br>○ Vrouw<br>○ Anders
Wie is uw Huisarts?	Naam:<br><br>Huisartsenpraktijk:<br><br>Plaatsnaam:
Indien van toepassing, bent u zwanger?	
○ Ja<br>○ Nee<br>○ Ik weet het niet	
Wat is uw lengte?	cm
Wat is uw gewicht?	kg
Wat is uw rookstatus?	
○ Ik rook niet en heb nooit gerookt<br>○ Ik heb vroeger gerookt maar ik ben helemaal gestopt<br>○ Ik rook af en toe, maar niet elke dag<br>○ Ik rook elke dag	
Welke van de onderstaande aandoeningen zijn op u van toepassing?	
○ Hoge bloeddruk<br>○ Suikerziekte type 1 (Diabetes Mellitus type 1)<br>○ Suikerziekte type 2 (Diabetes Mellitus type 2)<br>○ Hart- en vaatziekten (anders dan een hoge bloeddruk)<br>○ Obesitas<br>○ Te hoog cholesterol (vetstofwisselingsstoornis)<br>○ Bekend met te hoge hoeveelheid eiwit in de urine<br>○ Geen van de bovenstaande	

16.2 Appendix B: Guidelines for cardiovascular risk management ([Link to source document])

16.2.1 Cardiovascular risk profile in men ≥40 and women ≥50 years of age

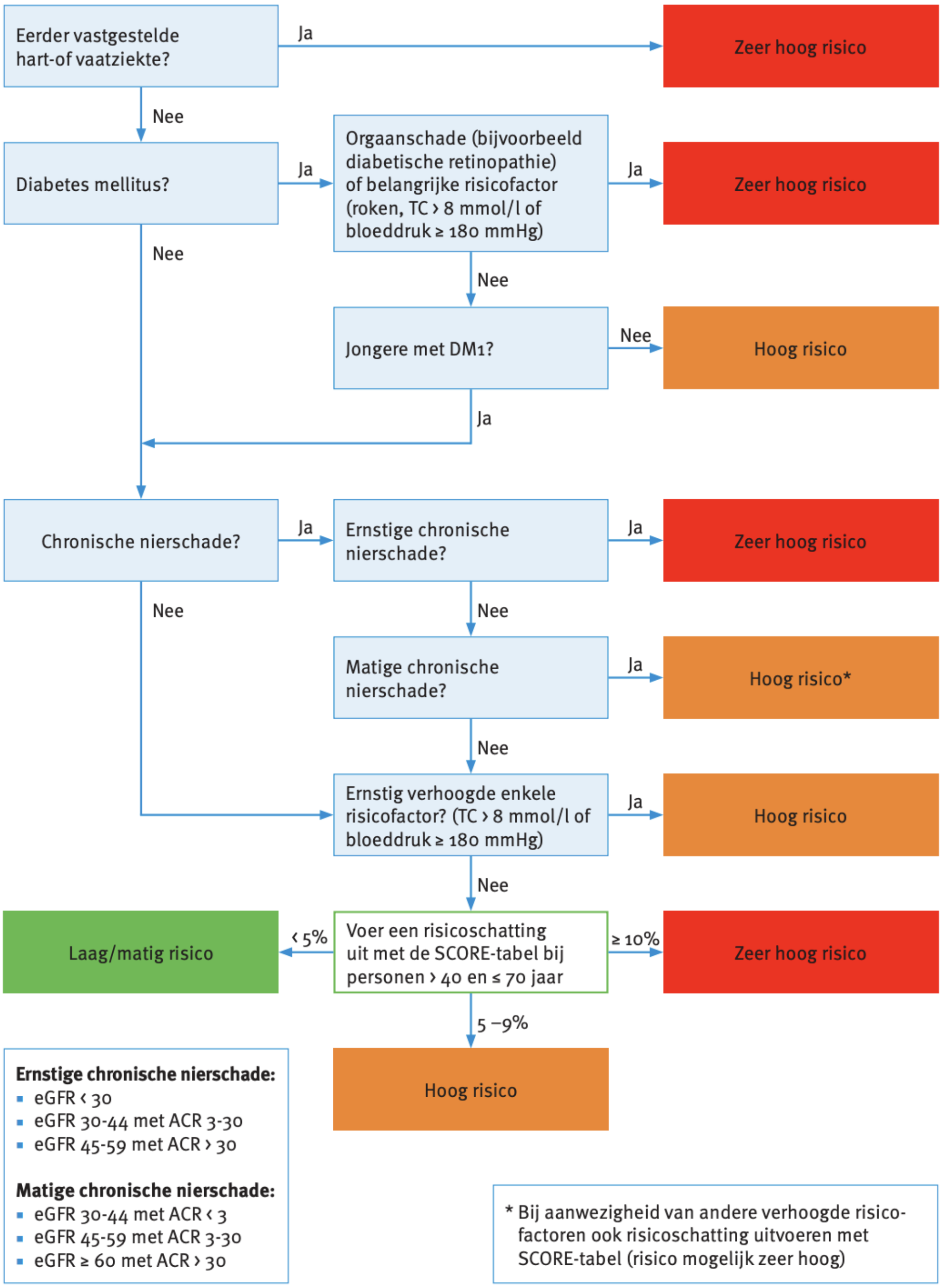

TC = totaal cholesterol; ACR: albumine-creatinineratio (mg/mmol)

16.2.2 Optimal treatment following the NHG guidelines

Zeer hoog risico | Hoog risico | Laag tot matig verhoogd risico

	Streefwaarde LDL-cholesterol (mmol/l)			Streefwaarde systolische bloeddruk (mmHg)**			Beleid
	≤ 70 jaar	> 70 jaar		≤ 70 jaar	> 70 jaar		
		Niet kwetsbaar	Kwetsbaar		Niet kwetsbaar	Kwetsbaar	
**Eerder vastgestelde hart- of vaatziekte** (onder andere acuut coronair syndroom; angina pectoris; coronaire revascularisatie; TIA of beroerte; aorta-iliofemorale atherosclerose; aorta-aneurysma; claudicatio intermittens of perifere revascularisatie). Bij beeldvorming aangetoonde atherosclerotische stenose of ischemie	< 1,8	< 2,6	Indien medicatie wordt gegeven bij voldoende levensverwachting: < 2,6	< 140 (eventueel < 130*)	< 150 (eventueel < 140*)	< 150 en ≥ 70 diastolisch	Leefstijladvies<br><br>Medicamenteuze therapie meestal aangewezen
**Diabetes mellitus met orgaanschade**, zoals proteïnurie, **of met een belangrijke risicofactor**, zoals roken of ernstige hypercholesterolemie (TC > 8 mmol/l) of ernstig verhoogde bloeddruk (≥ 180 mmHg)<br>**Ernstige chronische nierschade**: eGFR < 30 of 30-44 met ACR 3-30; eGFR 45-59 met ACR > 30<br>Tienjaarsrisico op sterfte aan HVZ met **SCORE ≥ 10%**	< 2,6	< 2,6 (overweeg medicatie bij voldoende levensverwachting)	Geen medicatie starten of stop lipidenverlagende medicatie	< 140 (eventueel < 130*)	< 150 (eventueel < 140*)	< 150 en ≥ 70 diastolisch	
**Matige chronische nierschade**: eGFR 30-44 met ACR < 3; eGFR 45-59 met ACR 3-30; eGFR ≥ 60 met ACR > 30<br>De meeste andere personen met **diabetes mellitus** die geen zeer hoog risico hebben<br>Ernstig verhoogde enkele risicofactor (TC > 8 mmol/l of bloeddruk ≥ 180 mmHg)<br>Tienjaarsrisico op sterfte aan HVZ met **SCORE ≥ 5% en < 10%**	< 2,6	< 2,6 (overweeg medicatie bij voldoende levensverwachting)	Geen medicatie starten of stop lipidenverlagende medicatie	< 140 (eventueel < 130*)	< 150 (eventueel < 140*)	< 150 en ≥ 70 diastolisch	Leefstijladvies<br><br>Overweeg medicamenteuze behandeling
Tienjaarsrisico op sterfte aan HVZ met **SCORE < 5%**. Veel personen van middelbare leeftijd vallen in deze categorie. **Jongeren met DM type 1 zonder klassieke risicofactoren**	n.v.t.	n.v.t.	n.v.t.	n.v.t.	n.v.t.	n.v.t.	Leefstijladvies aanbevolen<br><br>Medicamenteuze therapie zelden aangewezen

eGFR: estimated Glomerular Filtration Rate/geschatte nierfunctie; ACR: albumine-creatinineratio (mg/mmol); SCORE = Systematic Coronary Risk Evaluation; TC = totaal cholesterol.

* Indien medicatie om dit te bereiken verdragen wordt.

** De streefwaarden in deze tabel betreffen spreekkamermetingen.

Link to source document

16.2.3 SCORE table to estimate risk of cardiovascular mortality in the next 10 years

Vrouwen

Leeftijd	Bloeddruk	Niet-rookster						Rookster					
	TC-HDL-ratio	3	4	5	6	7	8	3	4	5	6	7	8
65	180	4	5	6	7	8	10	8	9	11	12	15	18
		15-18	18-21	21-25	25-30	30-36	37-44	30-34	33-40	39-46	46-54	>50	>50
	160	3	3	4	5	6	7	6	6	7	9	11	13
		11-13	13-15	15-18	18-21	21-26	26-31	20-21	24-28	28-33	33-39	40-47	48-58
	140	2	2	3	3	4	5	4	5	5	6	8	9
		8-9	9-10	10-12	13-15	15-18	19-22	14-17	17-20	20-23	23-28	28-34	35-42
	120	1	2	2	2	3	4	3	3	4	5	6	7
		5-6	6-7	7-9	9-11	11-13	13-16	10-12	12-14	14-17	17-20	20-24	25-30
60	180	2	3	3	4	5	6	4	5	6	7	8	10
		9-11	11-13	13-16	16-19	19-23	24-29	18-21	21-25	25-30	30-35	36-43	44-53
	160	2	2	2	3	3	4	3	3	4	5	6	7
		7-8	8-9	9-11	11-13	14-16	17-20	13-15	15-18	18-21	21-25	26-31	32-38
	140	1	1	2	2	2	3	2	2	3	4	4	5
		5-6	5-7	7-8	8-9	10-12	12-15	9-11	10-12	12-15	15-18	18-22	23-27
	120	1	1	1	1	2	2	1	2	2	3	3	4
		3-4	4-5	5-6	6-7	7-8	9-10	6-7	7-9	9-10	11-13	13-16	16-19
55	180	1	1	2	2	2	3	2	3	3	4	4	6
		6-7	7-9	9-10	10-13	13-16	16-20	11-14	13-17	16-20	20-24	24-29	30-37
	160	1	1	1	1	2	2	2	2	2	3	3	4
		4-5	5-6	6-7	7-9	9-11	11-14	8-10	9-12	11-14	14-17	17-21	21-26
	140	1	1	1	1	1	2	1	1	1	2	2	3
		3-4	4-4	4-5	5-6	6-8	8-10	6-7	7-8	8-10	10-12	12-15	15-19
	120	<1	<1	1	1	1	1	1	1	1	1	2	2
		2-3	2-3	3-4	4-5	4-6	6-7	4-5	5-6	6-7	7-9	9-11	11-13
50	180	1	1	1	1	1	1	1	1	1	2	2	3
		3-4	4-5	5-6	6-7	8-9	10-12	6-8	8-9	9-11	11-14	14-17	18-22
	160	<1	<1	1	1	1	1	1	1	1	1	2	2
		2-3	3-4	3-4	4-5	5-7	7-8	5-6	5-7	7-8	8-10	10-12	13-16
	140	<1	<1	<1	<1	1	1	<1	1	1	1	1	1
		2-2	2-2	2-3	3-4	4-5	5-6	3-4	4-5	5-6	6-7	7-9	9-11
	120	<1	<1	<1	<1	<1	1	<1	<1	<1	1	1	1
		1-1	1-2	2-2	2-3	3-3	4-4	2-3	3-3	3-4	4-5	5-6	6-8
45	180	<1	<1	<1	<1	<1	1	<1	<1	1	1	1	1
		2-2	2-2	2-3	3-4	4-5	5-6	3-4	3-4	4-5	5-7	7-9	8-11
	160	<1	<1	<1	<1	<1	<1	<1	<1	<1	1	1	1
		1-1	1-2	2-2	2-3	3-3	3-4	2-3	2-3	3-4	4-5	5-6	6-8
	140	<1	<1	<1	<1	<1	<1	<1	<1	<1	<1	<1	1
		1-1	1-1	1-1	1-2	2-2	2-3	1-2	2-2	2-3	3-3	3-4	4-5
	120	0	0	<1	<1	<1	<1	<1	<1	<1	<1	<1	<1
		1-1	1-1	1-1	1-2	1-2	2-2	1-1	1-2	2-2	2-2	2-3	3-4

Mannen

Leeftijd	Bloeddruk	Niet-roker						Roker						
	TC-HDL-ratio	3	4	5	6	7	8	3	4	5	6	7	8	
65	180	7	8	10	12	15	18	13	15	18	21	26	31	Sterfte
		22-28	26-33	31-39	37-48	46-58	>50	40-51	47-60	>50	>50	>50	>50	Ziekte + Sterfte
	160	5	6	7	9	11	13	9	11	13	16	19	23	Sterfte
		15-20	18-23	22-28	27-34	33-42	41-53	29-37	34-43	40-52	49-62	>50	>50	Ziekte + Sterfte
	140	3	4	5	6	8	10	7	8	9	11	14	17	Sterfte
		11-14	13-17	16-20	19-25	24-30	30-38	20-26	24-31	29-37	35-45	44-56	>50	Ziekte + Sterfte
	120	2	3	4	4	5	7	5	5	7	8	10	13	Sterfte
		8-10	9-12	11-14	14-18	17-22	22-28	14-18	17-22	21-27	25-32	32-40	39-50	Ziekte + Sterfte
60	180	4	5	6	8	10	12	8	10	12	15	18	22	Sterfte
		16-20	19-24	23-29	28-36	35-45	44-56	30-38	35-45	43-54	>50	>50	>50	Ziekte + Sterfte
	160	3	4	5	6	7	9	6	7	9	11	13	16	Sterfte
		11-14	14-17	16-21	20-26	25-32	32-40	21-27	25-32	31-39	37-47	46-58	>50	Ziekte + Sterfte
	140	2	3	3	4	5	6	4	5	6	8	9	12	Sterfte
		8-10	10-12	12-15	14-18	18-23	23-29	15-19	18-23	22-28	27-34	33-42	42-53	Ziekte + Sterfte
	120	2	2	2	3	4	5	3	4	4	5	7	9	Sterfte
		6-7	7-9	8-11	10-13	13-16	16-21	11-13	13-16	16-20	19-24	24-30	30-38	Ziekte + Sterfte
55	180	3	3	4	5	6	8	5	6	8	9	12	15	Sterfte
		11-13	13-16	16-20	19-25	24-31	30-39	19-25	24-30	29-37	36-45	44-56	>50	Ziekte + Sterfte
	160	2	2	3	4	5	6	4	4	5	7	8	11	Sterfte
		7-9	10-11	11-14	14-17	17-22	22-28	14-18	17-22	21-26	26-33	32-41	40-51	Ziekte + Sterfte
	140	1	2	2	3	3	4	3	3	4	5	6	8	Sterfte
		5-7	6-8	8-10	10-12	12-16	16-20	10-13	12-15	15-19	18-23	23-29	29-37	Ziekte + Sterfte
	120	1	1	1	2	2	3	2	2	3	3	4	5	Sterfte
		4-5	4-6	6-7	7-9	9-11	11-14	7-9	9-11	10-13	13-17	16-21	21-27	Ziekte + Sterfte
50	180	2	2	2	3	4	5	3	4	5	6	7	9	Sterfte
		6-8	8-10	10-12	12-15	15-19	20-25	12-16	15-19	18-23	23-29	28-36	36-46	Ziekte + Sterfte
	160	1	1	2	2	3	4	2	3	3	4	5	6	Sterfte
		4-6	6-7	7-9	9-11	11-14	14-18	9-11	10-13	13-16	16-20	20-26	26-33	Ziekte + Sterfte
	140	1	1	1	2	2	3	2	2	2	3	4	5	Sterfte
		3-4	4-5	5-6	6-8	8-10	10-13	6-8	7-9	9-12	11-15	15-18	19-24	Ziekte + Sterfte
	120	1	1	1	1	1	2	1	1	2	2	3	3	Sterfte
		2-3	3-4	3-4	4-6	6-7	7-9	4-5	5-7	7-8	8-10	10-13	13-17	Ziekte + Sterfte
45	180	1	1	1	2	2	3	2	2	2	3	4	5	Sterfte
		4-5	5-6	6-7	7-9	9-11	12-15	7-9	9-11	11-13	13-17	17-21	22-27	Ziekte + Sterfte
	160	1	1	1	1	2	2	1	1	2	2	3	4	Sterfte
		3-3	3-4	4-5	5-6	7-8	8-11	5-6	6-8	8-10	10-12	12-15	16-20	Ziekte + Sterfte
	140	<1	1	1	1	1	1	1	1	1	2	2	3	Sterfte
		2-2	2-3	3-4	4-5	5-6	6-8	4-4	4-5	5-7	7-9	9-11	11-14	Ziekte + Sterfte
	120	<1	<1	<1	1	1	1	1	1	1	1	1	1	Sterfte
		1-2	2-2	2-3	3-3	3-4	4-5	2-3	3-4	4-5	5-6	6-8	8-10	Ziekte + Sterfte

16.2.4 NHG Treatment recommendations lipid disorders

Therapieschema cholesterolbehandeling

Stap 1: statine

- Bij < 40% gewenste LDL-daling: geef atorvastatine 1 dd 10 mg (laagste kosten), rosuvastatine 1 dd 5 mg of simvastatine 1 dd 40 mg.
- Bij ≥ 40% gewenste LDL-daling: geef atorvastatine 1 dd 20 mg (laagste kosten) of rosuvastatine 1 dd 10 mg.
- Geef bij chronisch gebruik van CYP3A4-remmende of -inducerende middelen pravastatine 1 dd 40 mg.

Stap 2: intensiveer de lipidenverlagende therapie

- Indien de streefwaarde niet is bereikt, de patiënt geen (ernstige) bijwerkingen heeft en gemotiveerd is de therapie te intensiveren: verhoog de dosering in stapjes tot de maximale dosering (atorvastatine 1 dd 80 mg, rosuvastatine 1 dd 40 mg). Vervang simvastatine 40 mg bij onvoldoende effect door atorvastatine 10-20 mg of rosuvastatine 5-10 mg.

Stap 3: overweeg toevoegen ezetimib

- Indien de streefwaarde niet is bereikt: overweeg ezetimib 1 dd 10 mg toe te voegen bij patiënten mét hart- en vaatziekten ≤ 70 jaar. Weeg de voor- en nadelen van het toevoegen van ezetimib of het accepteren van een hogere LDL-waarde dan de streefwaarde samen af.
- Voor patiënten ≤ 70 jaar zonder hart- en vaatziekten kan toevoeging van ezetimib ook een optie zijn als voldoende LDL-reductie met een statine niet haalbaar blijkt. Weeg de voor- en nadelen van het toevoegen van ezetimib of het accepteren van een hogere LDL-waarde dan de streefwaarde samen af.
- Ouderen (> 70 jaar): overweeg toevoegen van ezetimib alleen bij niet-kwetsbare ouderen met hart- en vaatziekten.
- Overweeg verwijzing bij onvoldoende bereiken van de streefwaarde.

16.2.5 NHG Treatment recommendations hypertension

Table 3 NHG recommendations for treatment of hypertension

Situatie	Geneesmiddel
Verhoogde albuminurie (> 3 mg/mmol albumine/creatinine)	ACE-R/ARB
Eerder MI/coronairlijden	Bètablokkers, ACE-R/ARB
Angina pectoris	Bètablokkers, calciumantagonisten
Hartfalen	ACE-R/ARB, bètablokkers, diuretica, aldosteronantagonisten
Atriumfibrilleren	Bètablokkers, non-dihydropyridine-calciumkanaalblokkers, ACE-R/ARB, aldosteronantagonisten
Perifeer arterieel vaatlijden	ACE-R
Diabetes mellitus	ACE-R/ARB
Zwangerschap	Methyldopa, labetalol, calciumantagonisten
Personen van West- of Zuid-Afrikaanse afkomst	Diuretica en calciumantagonisten

ACE-R = angiotensineconverterende enzymremmer; ARB = angiotensinereceptorblokker; diuretica = thiazide of thiazideachtig; MI = myocardinfarct; non-dihydropyridine-calciumkanaalblokkers zijn verapamil en diltiazem

16.3 Appendix C: Medical Adherence Report Scale (MARS)

De Medication Adherence Report Scale – MARS test

De **Medication Adherence Report Scale (MARS)** is een meetinstrument voor therapietrouw waarbij 5 stellingen met de patiënt overlopen worden. Door het optellen van de score krijgt u een zicht op de therapietrouw: hoe hoger de score, hoe beter de therapietrouw. Bij een MARS-somscore van 21 of een score van 4 op elk individueel item wordt de patiënt als therapietrouw beschouwd.

	Altijd (score = 1)	Vaak (score = 2)	Soms (score = 3)	Zelden (score = 4)	Nooit (score = 5)
Ik vergeet mijn medicijnen te nemen	☐	☐	☐	☐	☐
Ik wijzig de dosering van mijn medicijnen	☐	☐	☐	☐	☐
Ik stop een tijdje met het innemen van mijn medicijnen	☐	☐	☐	☐	☐
Ik beslis een inname over te slaan	☐	☐	☐	☐	☐
Ik neem minder dan voorgeschreven	☐	☐	☐	☐	☐

MARS-vragenlijst[1]
